# Mannitol Stress Directs Flavonoid Metabolism toward Synthesis of Flavones via Differential Regulation of Two Cytochrome P450 Monooxygenases in *Coleus forskohlii*

**DOI:** 10.3389/fpls.2016.00985

**Published:** 2016-07-06

**Authors:** Praveen Awasthi, Ajai Prakash Gupta, Yashbir S. Bedi, Ram A. Vishwakarma, Sumit G. Gandhi

**Affiliations:** ^1^Indian Institute of Integrative Medicine (CSIR-IIIM), Council of Scientific and Industrial Research Jammu, India; ^2^Quality Control, Quality Assurance & CMC Division, Council of Scientific and Industrial Research-Indian Institute of Integrative Medicine Jammu, India; ^3^Division of Biological Science, Faculty of Science, Academy of Scientific and Innovative Research Kolkata, India

**Keywords:** 7-O-methylapigenin, CYP93B, CYP706C, genkwanin, naringenin

## Abstract

Cytochrome P450 monooxygenases (CYP450s) are known to play important roles in biosynthesis of all secondary metabolites, including flavonoids. Despite this, few CYP450s have been functionally characterized in model plants and roles of fewer CYP450s are known in non-model, medicinal, and aromatic plants. Our study in *Coleus forskohlii* indicates that flavone synthase (CYP93B) and flavonoid 3′ monooxygenase (CYP706C) are key enzymes positioned at a metabolic junction, to execute the biosynthesis of different sub-classes of flavonoids (flavones, flavonol, anthocynanin, isoflavones etc.) from a common precursor. Such branch points are favored targets for artificially modulating the metabolic flux toward specific metabolites, through genetic manipulation or use of elicitors that differentially impact the expression of branch point genes. Genkwanin, the only flavone reported from *C. forskohlii*, is known to possess anti-inflammatory activity. It is biosynthesized from the general flavonoid precursor: naringenin. Two differentially expressed cytochrome P450 genes (*CfCYP93B, CfCYP706C*), exhibiting maximum expression in leaf tissues, were isolated from *C. forskohlii*. Mannitol treatment resulted in increased expression of *CfCYP93B* and decrease in expression of *CfCYP706C*. Metabolite quantification data showed that genkwanin content increased and anthocyanin levels decreased in response to mannitol treatment. Alignment, phylogenetic analysis, modeling, and molecular docking analysis of protein sequences suggested that CfCYP93B may be involved in conversion of naringenin to flavones (possibly genkwanin *via* apigenin), while CfCYP706C may act on common precursors of flavonoid metabolism and channel the substrate toward production of flavonols or anthocynanins. Decrease in expression of CfCYP706C and increase in accumulation of genkwanin suggested that mannitol treatment may possibly lead to accumulation of genkwanin *via* suppression of a competitive branch of flavonoids in *C. forskohlii*.

## Introduction

Flavonoids are a diverse group of secondary metabolites with a ubiquitous presence in plants. Flavonoids are known to play significant roles in various aspects of plant biology, including UV-protection, floral pigmentation, plant defense, etc (Ferreyra et al., 2012). They are also well-known for their beneficial effects on human health (Mol et al., 1998; Winkel-Shirley, 2002; Bradshaw and Schemske, 2003). More than 10,000 flavonoids have been identified in plants (Dixon and Pasinetti, 2010). On the basis of structure, flavonoids can be classified into two main groups, 2-phenylchromanes (flavanones, flavones, flavonols, flavan-3-ols, and anthocyanidins), and 3-phenylchromanes (isoflavones, isoflavans, and pterocarpans; Whettena and Sederoffavb, 1995; Harmer, 2000; Winkel-Shirley, 2001). Flavonoids are synthesized through the common intermediate: naringenin (Dixon and Paiva, 1995). Cytochrome P450 monooxygenases (CYP450s) catalyze NADPH dependent hydroxylation of substrates and are involved in diverse metabolic pathways, including biosynthesis of flavonoids (Paul, 2005). FNS (CYP93B) converts flavonones (e.g., naringenin, eriodictyol, etc.) to flavones (e.g., apigenin, luteolin, tricetin, etc.) (Martens and Mithöfer, 2005). F3′M (CYP706C), which is closely related to flavonoid 3′,5′-hydroxylase (F3′5′H), was proposed to channelize the metabolic flux, using flavonones as precursors, toward biosynthesis of flavonol or anthocynanin (Koo et al., 2013). Increased expression of a F3′5′H resulted in increased hydroxylation of anthocyanidins, thereby an increased blue hue in the flower colors (Tanaka, 2006). Genes such as FNS and F3′M which are placed at a metabolic junction, serve as promising candidates for modulation of the metabolic flux, through use of elicitors or by genetic engineering, for increasing the flux through a specific branch of the pathway (Stephanopoulos, 1999). Flavone synthases are classified into two classes; FNS I (Flavone synthase I) and FNS II (Flavone synthase II). FNS I is a soluble enzyme which is classified as 2-oxoglutarate-dependent dioxygenase. In rice it has been shown that FNS I catalyzes the conversion of naringenin to apigenin using co-factors oxoglutarate, FeSO_4_, ascorbate, and catalase (Lee et al., 2008). FNS II, in contrast, is a membrane bound enzyme belonging to CYP93B family, which catalyzes the conversion of naringenin to apigenin, without the need of co-factors such as FeSO_4_, ascorbate, and catalase (Martens and Mithöfer, 2005). FNS II activity was first demonstrated in *Antirrhinum majus* L. (Plantaginaceae; Stotz and Forkmann, 1981) and osmotically stressed cell suspension culture of *Glycine max* (L.) Merr. (Fabaceae) (Kochs and Grisebach, 1987) and later was functionally characterized in wide range of plant species (Martens and Mithöfer, 2005).

*Coleus forskohlii* (Willd.) Briq. (Lamiaceae) is an important medicinal plant known for its pharmacological importance. *C. forskohlii* produces genkwanin (7-O-methylapigenin; a flavone) (Alasbahi and Melzig, 2010), which is an important non-glycosylated flavones found in herbs that possess anti-inflammatory properties. Genkwanin imparts this anti-inflammatory property to herbs through inhibition of proinflammatory mediators mainly *via* regulation of miR-101/MKP-1/MAPK pathway in LPS-activated macrophages (Gao et al., 2014). The biosynthesis of genkwanin likely involves the action of flavone synthase (Jeon et al., 2009). However, in *C. forskohlii* only few genes involved in secondary metabolism, such as geranylgeranyl diphosphate synthase (GGPPS), 1-deoxy-D-xylulose-5-phosphate reducto-isomerase (DXR), diterpene synthases, few CYP450s, and chalcone synthase have been studied (Engprasert et al., 2004, 2005; Zerbe et al., 2013; Awasthi et al., 2015, 2016a,b).

## Materials and methods

### Plant material and growth condition

*C. forskohlii* plants were collected from Bengaluru, India (12.9716°N, 77.5946°E) and identified by Dr. YS Bedi. A voucher specimen was submitted to Janaki Ammal Herbarium, Indian Institute of Integrative Medicine (IIIM) Jammu, India (Acronym RRLH, Accession no. 22164). Four week old plants of *C. forskohlii*, used for the expression study, were grown in green house under ambient lighting, in the month of October, 2015. Plants were watered once in a day (100 ml). Average relative humidity (RH%) and temperature were 71% and 24°C, respectively (meteorological data of Jammu region). Young leaves, mature leaves, stem, root, and root tips were collected, frozen in liquid nitrogen and stored at −80°C until RNA extraction. Demarcation between young and mature leaves of *C. forskohlii* is shown in Figure S1.

### RNA extraction and cDNA synthesis

Total RNA was isolated from different tissues of *C. forskohlii* using TRIzol® reagent (Invitrogen, Life Technologies, USA) according to the manufacturer's instruction and quantified using spectrophotometer (NanoDrop 2000c, Thermo Fisher Scientific, USA). Ten microgram of total RNA was treated with DNase (Ambion® TURBO DNA-*free*™, Life Technologies, USA). First strand cDNA synthesis was carried out using the ImProm-II™ Reverse Transcription System (Promega, USA) according to manufacturer's protocol, with an anchored oligo-dT_12_ primer (FirstChoice® RLM-RACE Kit, Ambion®, Life Technologies, USA) and 1 μg of DNase-treated RNA as a template.

### Isolation of differentially expressed CYP450s from *C. forskohlii*

Isolation of differentially expressed CYP450 transcripts from *C*. *forskohlii* was carried out using degenerate primers described previously (Awasthi et al., 2015). First strand cDNA was synthesized from leaves, stem, and roots. Further, these cDNAs were normalized using *actin* as an internal control. An equal quantity of normalized cDNA from different tissues was used as a template for primary PCR. Primary PCRs were carried out by using a primer (D1) designed from the conserved EEF(R)PER motif in combination with the 3′-RACE-outer-primer (FirstChoice® RLM-RACE Kit, Ambion®, Life Technologies, USA). The PCR product was diluted by a factor of 10^−1^ and used as a template for the corresponding secondary (nested) PCR reactions. Secondary PCRs were carried out by multiplexing three primers (FG1, FG2, and FG5) designed from the PFG motif with the 3′-RACE-inner-primer (FirstChoice® RLM-RACE Kit, Ambion®, Life Technologies, USA). Refer Table S1 for primer sequences and T_m_. Final PCR products were loaded on 6% PAGE. Differentially displayed bands were excised and purified using GenElute™ Gel Extraction Kit (Sigma-Aldrich, USA). These bands were further cloned and sequenced. Cloning and sequencing methods were followed as previously described (Awasthi et al., 2015).

### Cloning of full length *CfCYP93B* and *CfCYP706C*

The 3′ EST sequences, obtained in the previous step were, used to design the 5′ RACE primers for *CfCYP93B* and *CfCYP706C* (for primer detail see Table S1). 5′ end sequences of *CfCYP93B* and *CfCYP706C* were obtained using First Choice® RLM-RACE Kit (Ambion®, Life Technologies, USA).

GSP (Gene Specific Primer) CfCYP93B5R and 5′ RACE outer primer (provided in the kit), and GSP CfCYP93B5R and 5′ RACE inner primer (provided in the kit) were used for the primary and nested PCR reactions, respectively (Refer Table S1 for primer sequences). Thermal profile for nested PCR was as follows: 5 min at 95°C, 35 cycles of 30 s at 95°C, 30 s at T_m_ (Refer Table S1 for T_m_) and 2 min at 72°C followed by final extension at 72°C for 10 min. Amplified DNA fragment obtained from 5′ RACE reaction was cloned into pTZ57R/T plasmid (InsTAclone™ PCR Cloning Kit; Fermentas, Thermo Fisher Scientific, USA) and sequenced. The full length coding DNA sequence (CDS) of *CfCYP93B* was amplified using primers cdsCfCYP93BF and cdsCfCYP93BF (Table S1), designed from the sequence information of 3′ and 5′ RACE amplicons. All primers were obtained from Integrated DNA Technologies, USA. Similarly 5′ RACE fragment and full length CDS were cloned for CfCYP706C. See Table S1 for primer details.

### Sequences analysis

The nucleotide sequence of *CfCYP93B* and *CfCYP706C* were conceptually translated using the ExPASy translate tool (http://web.expasy.org/translate/). Using ORF Finder (http://www.ncbi.nlm.nih.gov/gorf/gorf.html) open reading frames of *CfCYP93B* and *CfCYP706C* were predicted. Predicted protein sequences of CfCYP706C and CfCYP93B were subjected to BLASTP analysis, to find out their homologs. Multiple sequence alignment of CfCYP706C and CfCYP93B with their respective homologs was carried out using CLC genomic workbench; CLC bio, a QIAGEN Company. Theoretical isoelectric point (pI) and molecular weight (Mw) of protein (http://web.expasy.org/compute_pi/) was calculated using Compute pI/Mw tool (Bjellqvist et al., 1993, 1994). Transmemebrane helices and topology of proteins were predicted using HMMTOP (http://www.enzim.hu/hmmtop/; Tusnády and Simon, 1998, 2001). The secondary structures of CfCYP93B and CfCYP706C were calculated using SOPMA(https://npsa-prabi.ibcp.fr/cgi-bin/npsa_automat.pl?page=/NPSA/npsa_sopma.html; Geourjon and Deléage, 1995). Conserved motifs and domains of CfCYP93B and CfCYP706C were assessed using CYP module of the cytochrome P450 engineering database (Fischer et al., 2006, 2007; Sirim et al., 2009, 2010). Phylogenetic analysis was carried out to cluster the CYP450s, isolated in this study, into their respective CYP450 families, as previously described (Awasthi et al., 2015). Briefly, protein sequences of four members from each of the major CYP450 families of flowering plants (CYP71-99, CYP701-723, CYP51) were downloaded from NCBI database. Sequences of CfCYP93B and CfCYP706C were clustered with the downloaded sequences and phylogenetic trees were constructed using the neighbor-joining method, in MEGA5 software (Tamura et al., 2011; Kumar et al., 2012). Tree topology support was assessed by bootstrap analysis (1000 replicates).

### Mannitol treatment

Four-week old plants of *C. forskohlii* were used for mannitol treatment. Mannitol was dissolved in 50 mL (500 mM) of 0.1% ethanol. Entire elicitor solution (50 mL) was sprayed on the aerial parts of potted plants, and samples were collected at five different time points: 0, 2, 5, 10, and 24 h. Plants sprayed with 50 mL of 0.1% ethanol served as control for elicitor experiment. Following these treatments, leaves were frozen in liquid nitrogen, and stored at −80°C. Subsequently, these samples were used for extraction of RNA. Similar experimental conditions have been used earlier for expression analysis of members of CYP86A subfamily in *Arabidopsis thaliana* (L.) Heynh. (Brassicaceae; Duan and Schuler, 2005). For genkwanin quantification, treated, and control plants were uprooted after 3 days of treatment and air dried.

### Semi-quantitative RT-PCR expression study

For RNA expression profiling of *CfCYP93B* and *CfCYP706C* in different tissues, first strand cDNA synthesis was carried out using DNase (Ambion® TURBO DNA-free™, Life Technologies, USA) treated total RNA and oligo dT primers (ImProm-II™ Reverse Transcription System; Promega, USA). cDNA was subjected to PCR using GSPs (gene specific primers) and *actin* primers (see Table S1 for primer sequences). *Actin* gene was used as a housekeeping internal control.

### Relative expression analysis using quantitative real-time RT-PCR (qPCR)

Extraction of RNA and cDNA synthesis was carried out as described above. Primers used for the qPCR study were designed using Primer3 software (http://primer3.ut.ee/). Primer details are given in Table S1. Before proceeding for qPCR, primers were tested using conventional end-point PCR for single band amplification and real-time PCR for a single peak in melting curve.

qPCRs were performed as described in previous studies (Awasthi et al., 2015; Rather et al., 2015). Each PCR reaction was carried out in triplicate and a non-template negative control was included. *Actin* gene was chosen as house-keeping internal control for normalization. The threshold cycle (C_t_) of the amplification curve was used for the calculations. The relative expression level was analyzed using the 2^−ΔΔct^ method (Livak and Schmittgen, 2001), where ΔΔ Ct = (C_t, target_ − C_t, actin_)_time x_ − (C_t, target_ − C_t, actin_)_time 0_.

### Homology modeling and docking studies

Protein models of CYP450s were prepared using homology modeling module of Schrödinger Suite 2013 (Maestro, version 9.4, Schrödinger, LLC, New York, NY, 2013.). Protein 3D structure were predicted using crystal structures of CYP17A1 (PDB ID: 3RUKA) and CYP1A2 (PDB ID: 2HI4A) as templates for CfCYP93B and CfCYP706C, respectively. Since crystal structures of CYP17A1 and CYP1A2 lack N-terminal transmembrane peptides of 30 and 33 amino acids, respectively, N-terminal transmembrane peptides (30 amino acids for CfCYP93B and 33 amino acids for CfCYP706C) were removed before protein 3D model preparation. Heme coordinates were copied from the respective templates of CfCYP93B and CfCYP706C. A covalent bond was created between the heme iron atom and the sulfur of the conserved axial cysteine. Oxygen atom was attached perpendicularly to the Fe center of heme plane and zero bond order was established and finally models were energy minimized using OLPS_2005 force field. To check the stereo chemical stability of protein model, Ramachandran plot were obtained from Procheck module of the SAVES server (Eisenberg et al., 1997; Wiederstein and Sippl, 2007; http://services.mbi.ucla.edu/SAVES/). For docking analysis, protein models were subjected to protein preparation wizard of Schrödinger Suite 2013 (Maestro, version 9.4, Schrödinger, LLC, New York, NY, 2013), The grid was generated around the heme plane of the protein model. Glide XP precision mode (Glide, version 5.9, Schrödinger, LLC, New York, NY, 2013) was used to carry out the molecular docking. Four probable substrate (flavanone) molecules (naringenin, butin, isosakuranetin, and eriodictyol) and five probable products (genkwanin, apigenin, leucopelargonidin, dihydrokaempferol, kaempferol) were used as ligand data set to carry out molecular docking studies for CfCYP93B and CfCYP706C. All the nine ligands were also checked for their drug like properties using lipinski conformation (http://www.scfbio-iitd.res.in/software/drugdesign/lipinski.jsp; Lipinski, 2004; Jayaram et al., 2012).

### Quantification of genkwanin by LC–ESI–MS/MS

Dried samples (leaves, stem and roots) were ground with a pestle and mortar. Powdered samples were extracted thrice using methanol for 3 h at 30°C. The methanol extracts were filtered and dried *in vacuo*. Selective detection and quatification of genkwanin in extracts was carried out using LC-ESI-MS/MS method *via* MRM (multiple reaction monitoring). For calibration of standard (genkwanin), stock solution of 1 mg/mL was appropriately diluted for making a five point calibration curve for genkwanin. The calibration equation [y = 1021.473342x − 15447.969250 (*r*^2^ = 0.991)] of components detected using LC-electrospray ionization (ESI) in negative-ion mode and quantified by chromatograms of MRM transition mass of 283/268 for genkwanin, was obtained by plotting LC–MS peak area (y) vs. the concentration (x, ng/mL) of calibrator. The equation showed very good linearity over the range (See File S1 for detail). The experiment was performed in triplicate and *p*-values were calculated.

### Anthocyanin quantification

Anthocyanin level was measured as per previously described protocol (Laby et al., 2000). Leaves from control and mannitol-treated plants were harvested after 3 days of treatment. Fresh leaves were weighed and then extracted with methanol: HCl (99:1) at 4°C. The OD_530_ and OD_657_ for each sample were measured and relative anthocyanin levels were determined using equation: OD_530_ − (0.25 × OD_657_) × Extraction volume (mL) × 1/weight of tissue sample (g) = relative unit of anthocyanin/g fresh weight of tissue. The experiment was perform in triplicate and *p*-values were calculated.

## Results

### Identification of differentially expressed CYP450s from *Coleus forskohlii*

**Figure 1:**
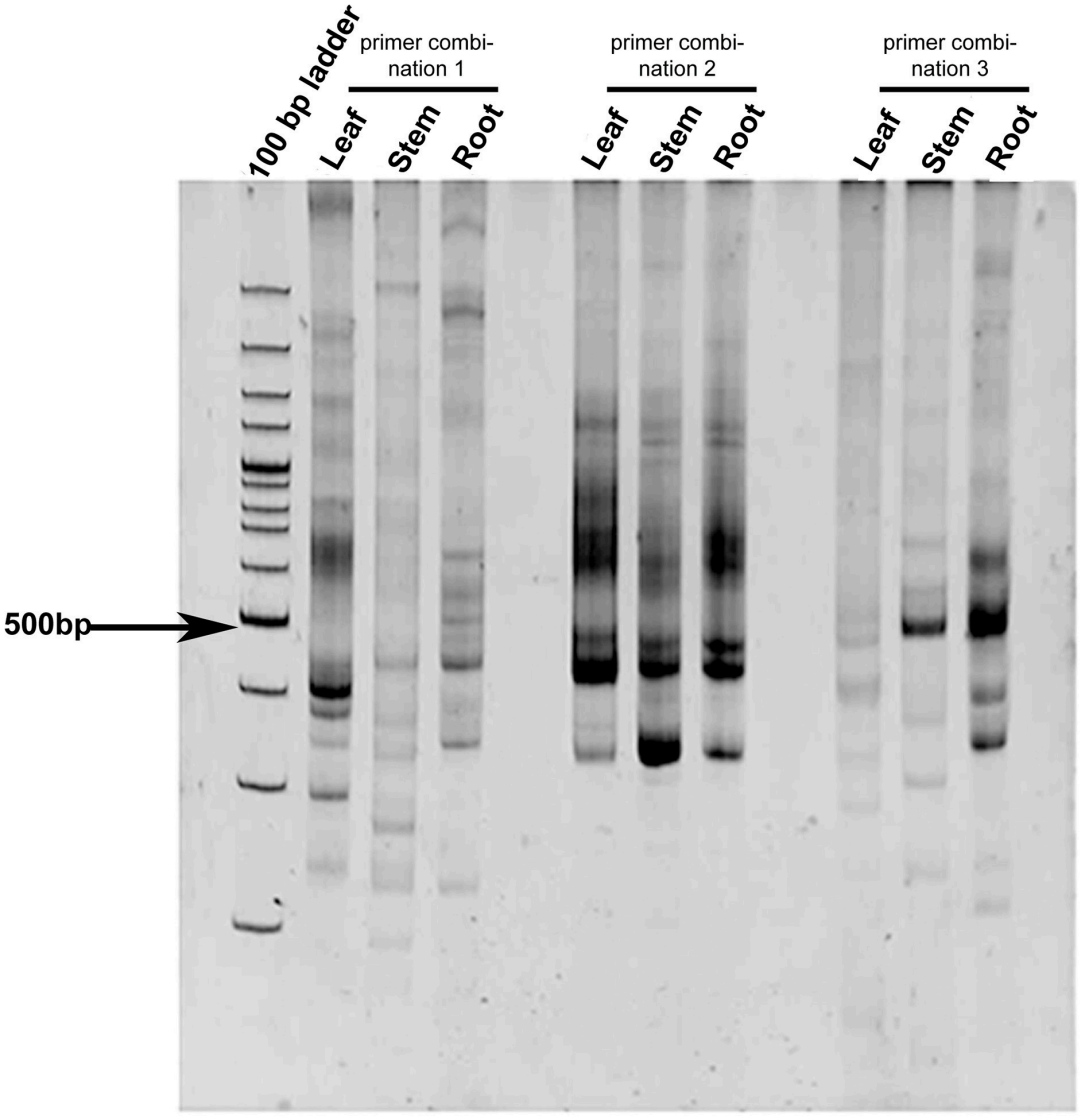
**Differential expression profile of CYP450 in ***C. forskohlii*****. The study was carried out in different tissues (leaf, stem, and root) of *C. forskohlii* using different degenerate primer combinations (designed from conserved domains of CYP450).

Leaf, stem, and root tissues of *C. forskohlii* were used for differential expression study of CYP450s from *C. forskohlii* (Figure 1). Differentially expressed PCR bands were excised from PAGE, cloned, and sequenced. Two gene sequences that were differentially expressed in leaves were identified as CYP450 on BLASTX analysis. These CYP450s were named on the basis of sequence identity with other members of various CYP450 families as; *CfCYP93B* and *CfCYP706C*. The amplicon size of *CfCYP93B* and *CfCYP706C* transcript fragments were 360 and 363 bp, respectively.

### Cloning and sequence analysis of CYP450s isolated from *C. forskohlii*

#### Cloning and sequence analysis of CfCYP93B

Sequence of *CfCYP93B* EST (360 bp) was used to design 5′ RACE primer for *CfCYP93B*. RACE-PCR was carried out to obtain the 5′ end of *CfCYP93B*, giving an amplicon size of 1470 bp. Full-length cDNA of *CfCYP93B* was 1776 bp in size, with ORF of 1530, 73 bp 5′ UTR, and 173 bp 3′ UTR (Figure S2). The full-length sequence of *CfCYP93B* was submitted to NCBI genbank database and accession ID (KF606861) was obtained. CfCYP93B encodes a protein of 509 amino acids, having a molecular weight of 57.542 kDa and theoretical pI 8.49. On BLASTX analysis, CfCYP93B was found to share maximum sequence identity of 85% with flavone synthase II (*Perilla frutescens Britton* var. *Crispa* Thunb.). Computational analysis showed the presence of an N-terminal signal peptide in CfCYP93B, which possibly targets the mature protein to endoplasmic reticulum (Figure S2). The secondary structure of CfCYP93B was predicted to contain 47.94% of α-helices, 4.72% of β-turns, 13.36% of extended strands, and 33.99% of random coils.

**Figure 2:**
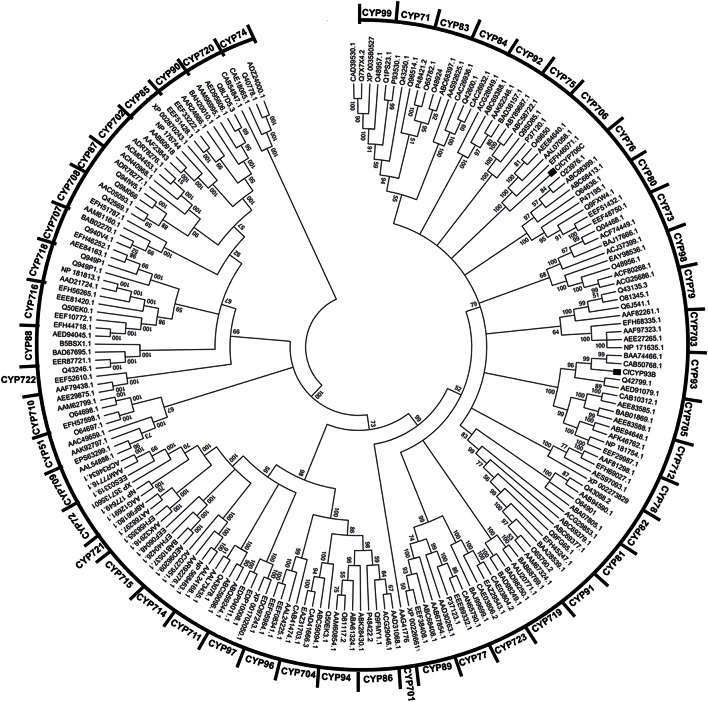
**Clustering of CfCYP93B and CfCYP706C from ***C. forskohlii*** with members from all major plant CYP450 families**. MEGA5 software was used for the analysis. Tree topology support was assessed by bootstrap analysis (1000 replicates).

CYP450s are well-conserved in structure but exhibit relatively less conservation in amino acid sequence. The conserved structure comprised of 11 α helices (labeled A–K) and 4 β-pleated sheets (labeled 1–4) that surround the buried catalytic site and contribute to overall fold (Graham and Peterson, 1999; Stout, 2004). Using the CYP module of the cytochrome P450 engineering database (Fischer et al., 2007), we analyzed the sequence of CfCYP93B. All the 11 α helices (labeled A–K) and 4 β-pleated sheets were found to be conserved in CfCYP93B. The (D/E)T pair and EXXR motif were present in I helix and K helix of CfCYP93B, respectively (Figure S2). Different homologs of CfCYP93B were identified using BLASTP, their sequences were downloaded from NCBI database and multiple sequence alignment was carried out. Cysteine heme-iron ligand signature (FGXGRRXCXG) was found to be conserved in these protein homologs (Figure S3). In phylogenetic tree analysis, CfCYP93B was found to cluster with members of the CYP93 family (Figure 2) belonging to A-TYPE CYP450s.

#### Cloning and sequence analysis of CfCYP706C

The 3′ EST sequence of *CfCYP706C* was used to design the 5′ RACE primer. RACE-PCR was carried out to obtain the 5′ end of *CfCYP706C*. The amplicon size obtained for the 5′ end of *CfCYP706C* was 1552 bp. Full-length cDNA of *CfCYP706C* was 1701 bp in size, with ORF of 1521 bp, 35 bp 5′ UTR, and 145 bp 3′ UTR (Figure S4). Full-length sequence of *CfCYP706C* was submitted to NCBI genbank database and accession ID (KC307774) was obtained. Protein encoded by *CfCYP706C* consisted of 506 amino acids with a molecular weight of 56.04 kDa and theoretical pI 8.14. On BLASTX analysis, CfCYP706C was found to have a maximum sequence identity of 79% with CYP706C35 [*Salvia miltiorrhiza* f. alba (Lamiaceae)]. An N-terminal signal peptide responsible for the localization of mature protein to endoplasmic reticulum was predicted in CfCYP706C sequence (Figure S4). The secondary structure of CfCYP706C comprised of 49.80% of α-helices, 6.32% of β-turns, 11.26% of extended strands, and 32.61% of random coils. All the 11 alpha helices and 4 beta sheets were found to be conserved in the structure of CfCYP706C. Homologs of CfCYP706C were identified using BLASTP tool and multiple sequence alignment was carried out. Cysteine heme-iron ligand signature (FGXGRRXCXG) was found to be conserved in these homologs (Figure S5). CfCYP706C was found to be A-TYPE CYP450 that clustered with the members of CYP706 family (Figure 2).

### Expression study

#### Expression analysis of CfCYP93B, CfCYP706C in tissues

**Figure 3:**
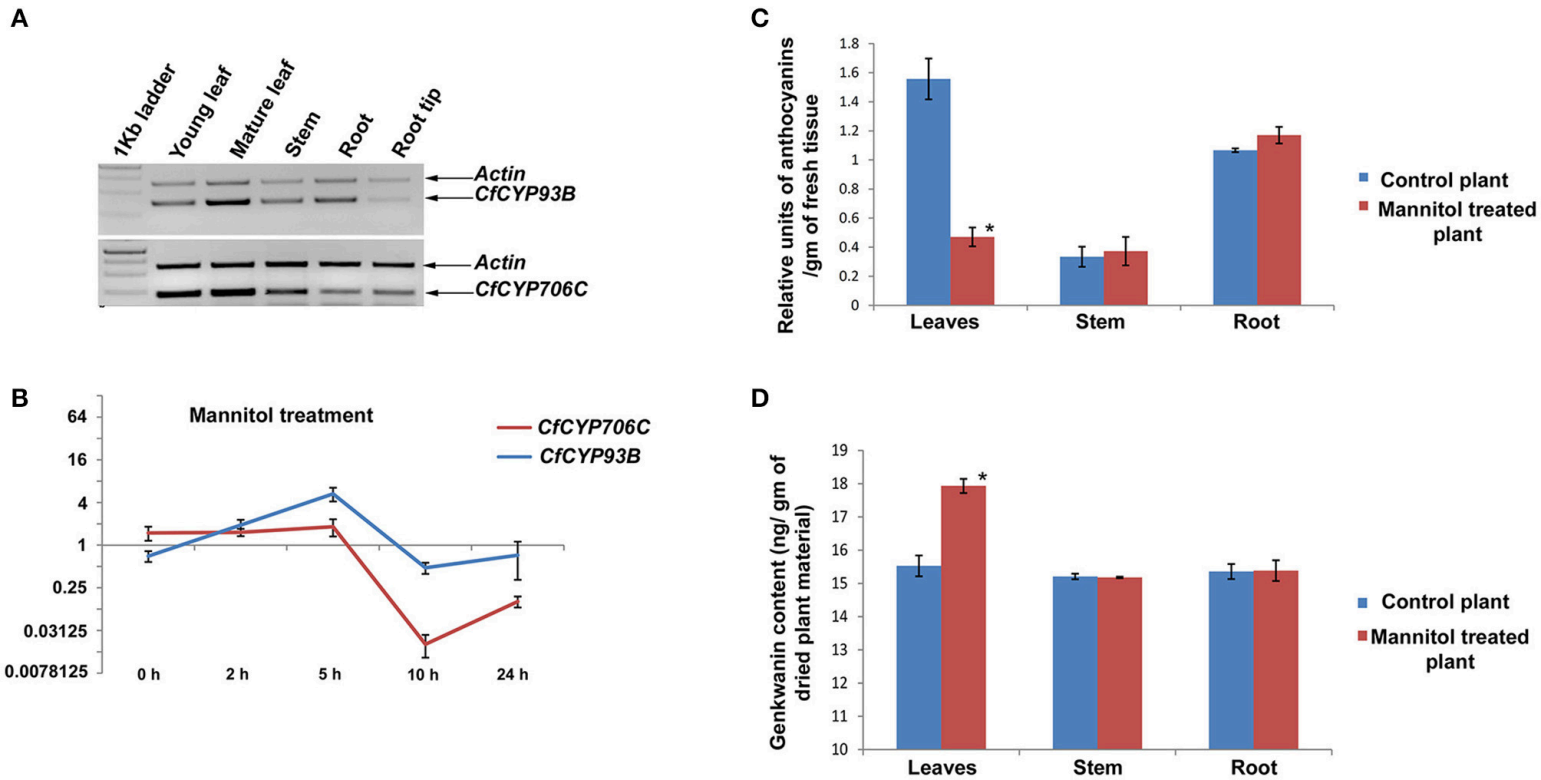
**Expression study of CfCYP93B and CfCYP706C and its correlation with genkwanin and anthocyanin content**. **(A)** Semi quantitative RT-PCR expression study of CfCYP93B and CfCYP706C in different tissues (young leaves, mature leaves, stems, roots, and root tips) of *C. forskohlii*. **(B)** qPCR study of CfCYP93B and CfCYP706C in response to mannitol treatment at different time interval (0, 2, 5, 10, and 24 h). Actin was used as housekeeping gene. For qPCR study, *p* < 0.05. **(C)** Relative anthocyanin units in different tissues (leaves, stem, and root) in response to mannitol stress. **(D)** Genkwanin content in different tissues (leaf, stem, and root) in response to mannitol stress. ^*^ indicates *p* < 0.05.

Expression analysis of CfCYP93B and CfCYP706C was carried out using semi-quantitative RT-PCR from young leaf, mature leaf, stem, root, and root tip of *C. forskohlii*. *Actin* was used as an internal control for normalization. CfCYP93B showed the highest expression in young leaves followed by mature leaves, root, root tip, and stem. Whereas, CfCYP706C expression was dominant in young leaves and mature leaves (Figure 3A).

#### qPCR analysis of CfCYP93B and CfCYP706C under mannitol treatment

Time course expression profiles of *CfCYP93B* and *CfCYP706C* in response to mannitol treatment were studied using quantitative real-time RT-PCR. *Actin* was used as housekeeping control for normalization and relative quantification was carried out by taking the expression of the gene of interest at 0 h (just before treatment) as a baseline for calculating fold change. *CfCYP93B* was upregulated in response to mannitol treatment. We observed that *CfCYP93B* mRNA accumulation reached maximum level after 5 h of treatment and then gradually reduced to almost the same levels as found in untreated plants after 10 h of treatment. *CfCYP706C* was down-regulated in response to mannitol treatment. *CfCYP706C* expression reached to its *minima* after 10 h of treatment and then its expression level gradually increased almost to its base level expression after 24 h of mannitol treatment (Figure 3B).

### Homology modeling and docking studies

#### Protein modeling and docking studies of CfCYP93B and CfCYP706C

Homology modeling was carried out for CfCYP93B and CfCYP706C using CYP17A1 (PDB ID: 3RUKA) and CYP1A2 (PDB ID: 2HI4A) as templates, respectively. Protein models were developed using Schrödinger suite 2013 (Maestro, version 9.4, Schrödinger, LLC, New York, NY, 2013). Heme groups copied from the templates were incorporated in the respective protein models (Figures S6A, S6B). Loop refinement of the protein models (CfCYP93B and CfCYP706C) was carried out using Prime module of Schrödinger Suite 2013 (Maestro, version 9.4, Schrödinger, LLC, New York, NY, 2013.). Total potential energy of CfCYP93B and CfCYP706C before loop refinement was calculated to be −7.9 × 10^4^ Kcal/mol and −8.04 × 10^4^ Kcal/mol, respectively. After loop refinement potential energy of CfCYP93B and CfCYP706C was found to be reduced to −9.0 × 10^4^ and −9.10 × 10^4^ Kcal/mol, respectively. The protein models were then energy minimized using force field (OPLS2005) module of protein preparation wizard (Maestro, version 9.4, Schrödinger, LLC, New York, NY, 2013). Total potential energy after energy minimization step, for CfCYP93B and CfCYP706C, was −9.1 × 10^4^ and −9.21 × 10^4^ Kcal/mol, respectively. Root mean square deviations (RMSD) for CfCYP93B and CfCYP706C with respect to their respective templates, after loop refinement and energy minimization step, were 0.933443 Å and 0.98722 Å, respectively. The stereo-chemical analysis of the predicted models of CfCYP93B and CfCYP706C proteins was analyzed by PROCHECK server (http://services.mbi.ucla.edu/SAVES/). Ramachandran plot analysis of CfCYP93B showed 83.7% residues in the most favorable region, 13.9% residues in the additional allowed region, 1.0% in the generously allowed region and 1.4% in the disallowed region (Figure S7A). Whereas, CfCYP706C model, showed 84.3% residues in the most favorable region, 12.1% residues in the additional allowed region, 2.5% residues in the generously allowed region and 1.0% in the disallowed region (Figure S7B). The results of the PROCHECK analysis indicated that a relatively low percentage of residues had phi/psi angles in the disallowed regions suggesting the acceptability of Ramachandran plots for the modeled CYP450s proteins.

**Figure 4:**
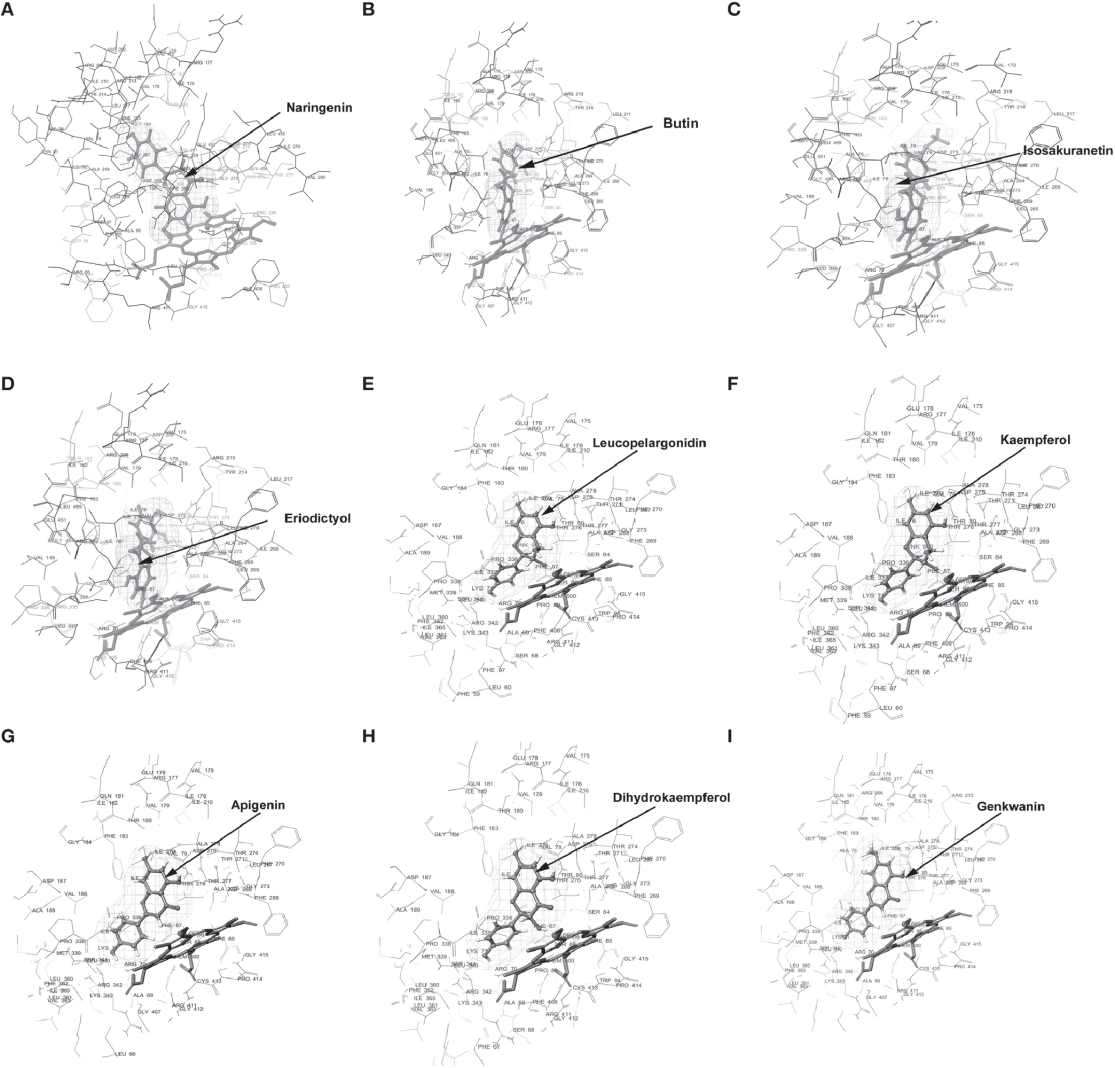
**Molecular docking of CfCYP93B**. Diagram showing **(A)** Naringenin, **(B)** Butin, **(C)** Isosakuranetin, **(D)** Eriodictyol, **(E)** Leucopelargonidin, **(F)** Kaempferol, **(G)** Apigenin, **(H)** Dihydrokaempferol, **(I)** Genkwanin, docked in the proposed binding pocket (above heme plane) of CfCYP93B. Mesh represents the ligand surface.

**Figure 5:**
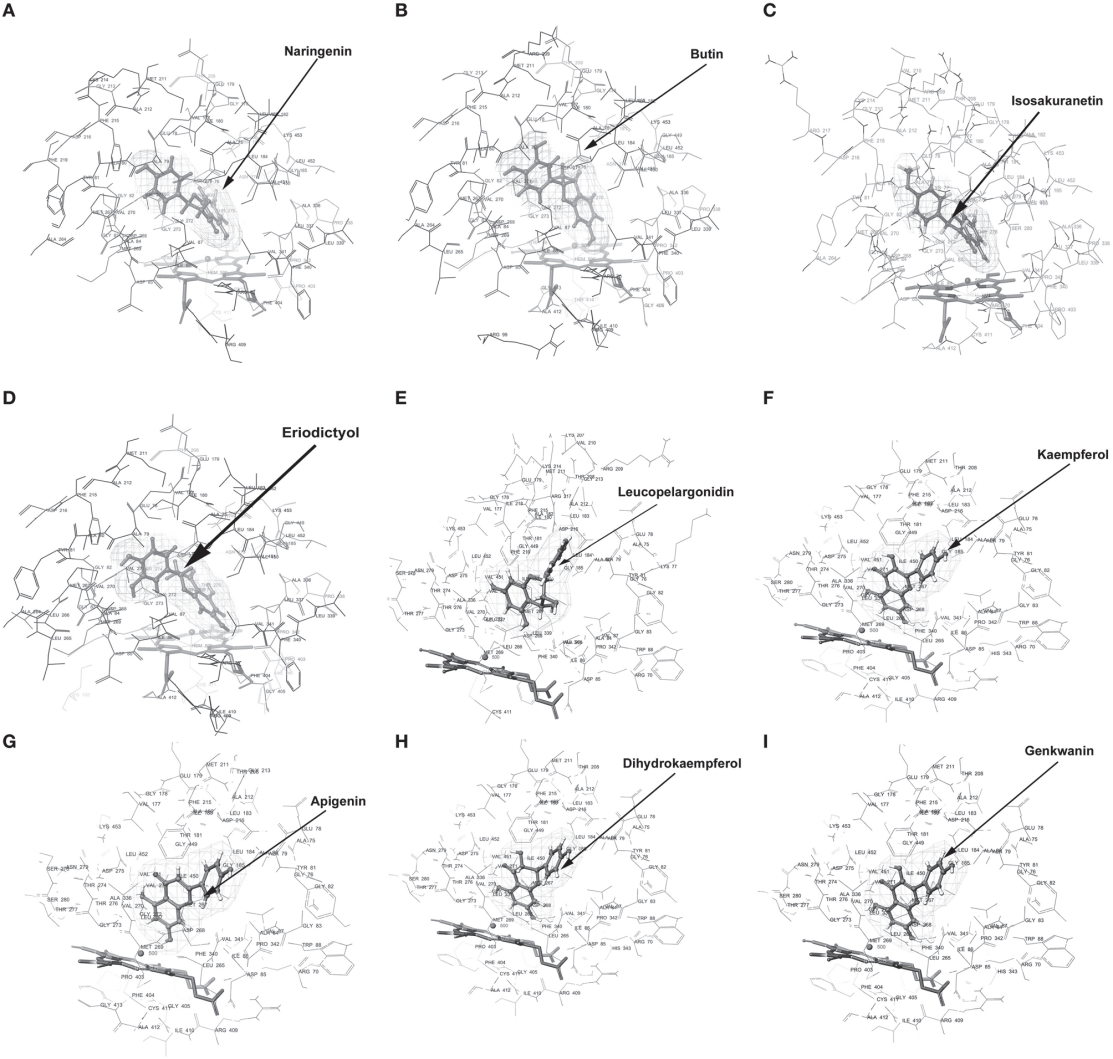
**Molecular docking of CfCYP706C**. Diagram **(A)** Naringenin, **(B)** Butin, **(C)** Isosakuranetin, **(D)** Eriodictyol, **(E)** Leucopelargonidin, **(F)** Kaempferol, **(G)** Apigenin, **(H)** Dihydrokaempferol, **(I)** Genkwanin, docked in the proposed binding pocket (above heme plane) of CfCYP706C. Mesh represents the ligand surface.

Docking experiments were performed using flavanones (naringenin, butin, isosakuranetin, and eriodictyol) as ligand data set (probable substrate) for CfCYP93B and CfCYP706C protein models. CfCYP93B showed a comparatively higher binding affinity for naringenin and eriodictyol. Eriodictyol and naringenin were found to be comfortably placed in the proposed binding pocket (above the heme plane) as shown in Figure 4. Docking data showed that ASP 268 was involved in hydrogen bond formation with butin and eriodictyol with a bond length of 2.24 and 2.16 Å, respectively (Figure S8), suggesting that ASP 268 (CfCYP93B) could play an important role in substrate binding. Based on the docking score, CfCYP706C showed a similar binding affinity for all the ligands i.e., naringenin, butin, isosakuranetin, and eriodictyol (Table 1). All the four ligands were quite suitably placed above the heme plane as shown in Figure 5. It is proposed that ASP 275 (1.66 Å) may be involved in hydrogen bond formation with naringenin and isosakuranetin while ASP 268 (2.36 Å) may be involved in hydrogen bond formation with eriodictyol (Figure S9). ASP 268 (2.36 Å) and ASP 275 (1.66 Å) could be important for substrate binding in CfCYP706C.

**Table 1:** **Docking scores of ligands with CfCYP93B and CfCYP706C**.

**S. no**	**Ligand**	**Docking score**		**G-energy (Kcal/mol)**	
		**CfCYP93B**	**CfCYP706C**	**CfCYP93B**	**CfCYP706C**
1	Naringenin	−4.273103	−8.35417	−9.0087	−28.0330
2	Isosakuranetin	−2.570103	−8.655886	−11.3733	−11.3733
3	Eriodictyol	−3.649534	−8.817555	−6.8596	−37.4519
4	Butin	−3.164433	−8.779972	−5.6604	−25.3422
5	Genkwanin	−**9.20221**	−6.820176	−40.7536	−31.5550
6	Apigenin	−8.441807	−8.436127	−36.4785	−39.1958
7	Leucopelargonidin	−8.822537	−**11.449823**	−24.6823	−39.0709
8	Dihydrokaempferol	−7.96974	−8.395646	−25.6895	−36.6680
9	Kaempferol	−7.64863	−8.835098	−31.9239	−39.3400

*Bold values indicate highest docking score among the ligands under study*.

Molecular docking was also carried out using apigenin, genkwanin, leucopelargonidin, dihydrokaempferol, and kaempferol as ligand dataset (probable products) for CfCYP93B and CfCYP706C (Figures 4, 5). CfCYP706C exhibited highest binding affinity (−11.449823) for leucopelargonidin (Table 1) which revealed that CfCYP706C may be involved in biosynthesis of anthocyanins. ASP 275 and ALA 80 were found to be involved in hydrogen bond formation with ligand leucopelargonidin (Figure S9). CfCYP93B had highest binding affinity for genkwanin. ASP 275 and SER 314 residues were found to be involved in hydrogen bond formation with genkwanin (Figure S8). These nine ligands were also checked for drug like properties (Table S2).

#### Metabolic profiling

Mannitol treatment of *C. forskohlii* plants resulted in an increase in expression of CfCYP93B and decrease in expression of CfCYP706C. Modeling and docking analysis revealed that these two genes may be involved in channeling the common substrate (product of phenylpropanoid pathway) into two different branches of flavonoids. In order to understand what implications the inversely correlated expression of these two CYP450s would have on metabolic profile, quantification of anthocyanin, and genkwanin was carried out in different tissues (leaves, stem, and roots) of mannitol treated *C. forskohlii* plants. The experiments were performed in triplicates and *p*-values were calculated to assess the statistical significance of data. Total anthocyanin level was found to be appreciably decreased (69.76%, with respect to control) in leaves of the mannitol-treated plants, whereas, there was no significant change in anthocyanin levels in stem and roots (Figure 3C). In contrast, genkwanin content was found to be considerably increased (15.49%, with respect to control) in mannitol-treated leaves, whereas, there was no noteworthy change in genkwanin content in the stem and root tissues (Figure 3D). Data was statistically validated through *t*-test.

## Discussion

CYP450s play an important role in the biosynthesis of secondary metabolites (Bak et al., 2011). Not many CYP450s have been functionally characterized in model plants and roles of even fewer have been indicated in non-model plants that produce medicinal compounds with important pharmacological activities (Zerbe et al., 2013; Guo et al., 2014). Few small (3–4 amino acids residues) motifs (EXXR, PERF, PFG motifs) are conserved among different families of CYP450s (Schopfer and Ebel, 1998), making the cloning of CYP450s, using homology-based approach a difficult task. Here, we have used a degenerate primer based differential expression study to isolate CYP450s from *C. forskohlii*. Previously, differential display study has been used in various plants (*Glycine max* (L.) Merr. (Fabaceae), *Gerbera hybrida* cv. (Asteraceae), *Astragalus chrysochlorus* Boiss and Kotschy (Fabaceae)) to isolate differentially expressed CYP450 transcripts (Schopfer and Ebel, 1998; Martens and Forkmann, 1999; Schoendorf et al., 2001; Turgut-kara and Ari, 2011). Differential display was found to be useful in the identification of key CYP450s involved in the biosynthesis of taxol in *Taxus cuspidata* Siebold and Zucc. (Taxaceae; Schoendorf et al., 2001).

Two CYP450s isolated from *C. forskohlii* were found to be clustered with members of CYP93 and CYP706 families and so they were named CfCYP93B and CfCYP706C, respectively. CYP93B family is generally involved in the biosynthesis of flavonoids (Akashi et al., 1999; Ayabe and Akashi, 2006). CYP93B isolated from *Gerbera hybrida* encodes a flavones synthase II, which catalyzes the direct formation of flavones from flavanones (Martens and Forkmann, 1999). BLASTP analysis revealed that CfCYP706C had considerable sequence identity with flavonoid 3′ monooxygenases from other plants, such as *Sesamum indicum* Burm. (Pedaliaceae; 74% identify, *e*-value 0.0) and *Solanum lycopersicum* (70% identify, *e*-value 0.0). Till date, there has been no report of functional characterization of any CYP706C member. None of the seven genes of CYP706 family in the *Arabidopsis* genome have been functionally characterized, so far (Bak et al., 2011). Only one report in *Zingiber officinale* Roscoe (Zingiberaceae) suggests that CYP706C may be closely related to flavonoids 3′ 5′ hydroxylase (Koo et al., 2013). One member of another subfamily (CYP706B) from *Gossypium hirsutum* L. (Malvaceae) in known to be involved in the biosynthesis of terpenoids (Luo et al., 2001; Bak et al., 2011).

*CfCYP706C* and *CfCYP93B* were both found to be dominantly expressed in leaves of *C. forskohlii*. However, they exhibited opposite expression patterns in response to mannitol treatment. *CfCYP93B* was found to be upregulated, while *CfCYP706C* was downregulated on mannitol treatment. Flavone synthase genes (GmFNSII-1 and GmFNSII-2) from *Glycine max* were also reported to be induced by mannitol treatment (Yan et al., 2014). To understand the role of CYP450s and prediction of their substrate and product profiles, protein modeling, and docking was found to be a promising approach (Rupasinghe and Schuler, 2006). Protein modeling can be carried out using different approaches such as, homology modeling, threading, and *ab intio*. The proteins that we have studied (CfCYP93B and CfCYP706C) are 509 and 506 amino acid residues long. The accuracy of protein model generated through *ab initio* approach is generally lower and it work betters for smaller proteins with lesser than 100 amino acid residues (Lee et al., 2009). Docking of substrates and evaluation of binding strategies of four CYP450s involved in biosynthesis of lignin, flavonoids, and anthocyanins was carried out using homology modeled proteins (Rupasinghe et al., 2003). Another study (Zhang et al., 2012), where modeling of 279 CYP450s (from *Arabidopsis thaliana*) longer than 300 amino acid residues was carried out and a database was created, showed that the models generated through homology based approach were better than those generated using threading and were well-suited for docking studies. Homology modeling and docking of CfCYP93B and CfCYP706C was carried out with two different ligand datasets: probable substrates and products. Amongst four flavonones that may act as probable substrates for CfCYP93B, it showed highest affinity for naringenin, which is the general precursor for most flavonoids. Important amino acid residues (CfCYP93B and CfCYP706C) which were found to be interacting with different ligands are enlisted in Table S3. Clustering and family classification also supports this analysis as other members of CYP93B are also known to use naringenin as the substrate (Martens and Mithöfer, 2005). On the other hand, CfCYP706C appeared to show almost similar affinity for the four possible substrate flavonones that were tested, suggesting that it may have a promiscuous activity. As such, some of the CYP450s involved in plant secondary metabolism are known to exhibit promiscuity in their substrate profiles (Wellmann et al., 2004; Jung et al., 2011). Further, there is no information available regarding the substrate profiles of other members of CYP706C family. Amongst possible products, CfCYP93B and CfCYP706C were found to have highest docking score for genkwanin and leucopelargonidin, respectively. Leucopelargonidin is a compound related to anthocyanins. Earlier report (Koo et al., 2013) indicates that CYP706C members may be involved in biosynthesis of flavonols or anthocyanins, using flavanones as substrates. However, our docking analysis shows that CfCYP70C exhibits much less affinity for flavonols as compared to anthocyanin related compounds (leucopelargonidin). Naringenin when acted upon sequentially by naringenin 3-dioxygenase and a dihydroflavonol 4-reductase, leads to production of leucopelargonidin *via* an intermediate dihydrokaempferol (Jung et al., 2011; Tanaka and Brugliera, 2013). Butin, another possible substrate taken in our study, is generally converted to 5-deoxyleucocyanidin (related to anthocyanins) *via* an intermediate dihydrofisetin, through sequential action of naringenin 3-dioxygenase, and a dihydroflavonol 4-reductase (Kanehisa and Goto, 2000). Eriodictyol may be converted to catechins or leucocyanidin/cyanidins in multistep reactions (Liu et al., 2015).

**Figure 6:**
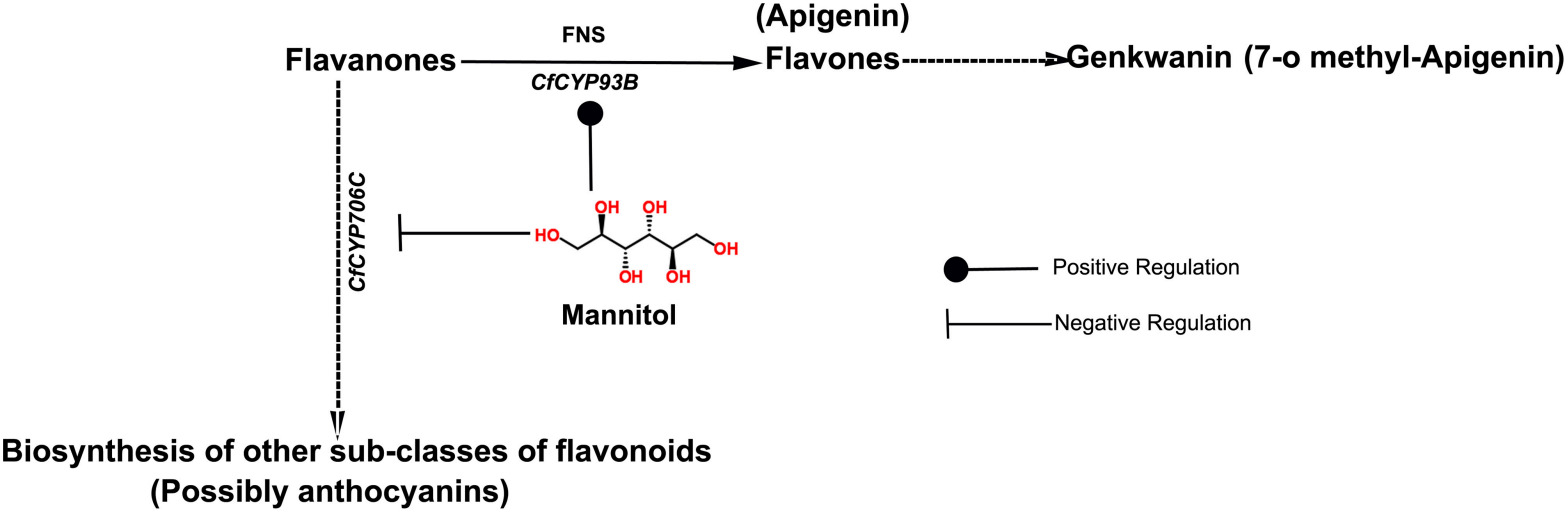
**Regulation of transcript expression of CfCYP93B and CfCYP706C in response to mannitol treatment**. Positive and Negative Regulation of CfCYP93B and CfCYP706C in response to mannitol treatment, respectively.

Interestingly, metabolic profiling revealed that genkwanin content increased significantly and anthocyanin level was notably decreased in leaves of *C. forskohlii* plants, on mannitol treatment (Figure 6). This is congruent with the expression profiles and likely product profiles of CfCYP93B and CfCYP706C, respectively. These results suggest that *CfCYP93B* and *CfCYP706C* are possibly placed at a metabolic junction, utilizing common precursor(s) generated from the phenylpropoanoid pathway and that they (*CfCYP93B* and *CfCYP706C*) are inversely regulated on mannitol treatment (Figure 6). On treatment with mannitol, precursor from phenylpropanoid pathway is possibly diverted toward biosynthesis of flavones (genkwanin) with concomitant decrease in the products of competitive pathwaysthat yield other sub-classes of flavonoids (anthocyanins).

## Author contributions

PA carried out the experimental work and prepared the first draft of manuscript and figures. AG carried out the quantification of genkwanin. YB and RV provided critical inputs for the study as well as during preparation of the manuscript. SG designed the study, analyzed the results, and edited the manuscript and figures.

### Conflict of interest statement

The authors declare that the research was conducted in the absence of any commercial or financial relationships that could be construed as a potential conflict of interest.

## Supplementary material

Figure S1 **Pictorial representation of demarcation between young and mature leaves of ***Coleus forskohlii*****.

Figure S2 **Nucleotide sequence analysis of CfCYP93B. 5′ and 3′ UTR region are shaded with light gray color**. Start codon is shaded with yellow color and stop codon is marked as asterisk (^*^) sign. Transmembrane helix region is underlined. ORF start from nucleotide number 74–1603. Meander loop is shaded dark red, heme domain (FGXGRRXCXG) shaded violet. DT pair and EXXR motif are shaded green and blue, respectively.

Figure S3 **Multiple sequence alignment of CfCYP93B with other homologs**. Conserved residues are shaded with dark red color. Amino acid sequence with the Cytochrome P450 cysteine heme-iron ligand signature (FGXGRRXCXG) is underlined. *Coleus forskohlii* (Accession No. KF606861); *Ocimum basilicium* (Accession No. AGF30365.1); *Perilla frutescens* (Accession No. BAB59004.1); *Salvia miltiorrhiza* (Accession No. AJD25217.1); *Picrorhiza kurrooa* (Accession No. AEG73887.1).

Figure S4 **Nucleotide sequence analysis of CfCYP706C. 5′ and 3′ UTR region are shaded with light gray color**. Start codon is shaded with yellow color and stop codon is marked as asterisk (^*^) sign. ORF start from nucleotide number 36–1556. Transmembrane helix region is shaded with pink color, Meander loop is marked with bright green color (DSPTEFIPER), heme domain (FGXGRRXCXG) shaded violet, DT pair is marked with turquoise color, EXXR, and WXXXR motif are shaded dark blue and dark red, respectively.

Figure S5 **Multiple sequence alignment of CfCYP706 with other homologs**. Conserved residues are shaded with dark red color. Amino acid sequence with the Cytochrome P450 cysteine heme-iron ligand signature (FGXGRRXCXG) is underlined. *Coleus forskohlii* (Accession No. KC307774); *Salvia miltiorrhiza* (Accession No. AJD25237.1); *Solanum lycopersicum* (Accession No. XP_004245721.1); *Vitis vinifera* (Accession No. XP_002263860.2); *Glycine max* (Accession No. XP_003549879.1).

Figure S6 **Protein 3D structure and conserved motif prediction of CfCYP93B and CfCYP706C. (A)** 3D structure representation of CfCYP93B and **(B)** 3D structure representation of CfCYP706C as ribbon display 3D structures, predicted by homology modeling module of Schrödinger Suite 2013. Predicted conserved motifs (EXXR motif, heme motif, meander loop, and DT pair) are marked in protein (CfCYP93B and CfCYP706C).

Figure S7 **Ramachandran plot of two CYPs**. Ramachandran plot of CfCYP93B **(A)** and CfCYP706C **(B)**. The plot calculations for the 3D models of CYPs proteins were computed with the PROCHECK server. Most favored regions are colored red (A, B, L), additional allowed (a, b, l, p), generously allowed [,a, ,b, ,l, ,p), and disallowed regions are indicated as yellow, light yellow, and white regions, respectively.

Figure S8 **2D Representation of interaction between CfCYP93B and different ligand dataset. (A)** Naringenin, **(B)** Butin, **(C)** Isosakuranetin, **(D)** Eriodictyol, **(E)** Leucopelargonidin, **(F)** Kaempferol, **(G)** Apigenin, **(H)** Dihydrokaempferol, **(I)** Genkwanin. Arrow indicates hydrogen bond.

Figure S9 **2D Representation of interaction between CfCYP706C different ligand dataset. (A)** Naringenin **(B)** Butin **(C)** Isosakuranetin **(D)** Eriodictyol **(E)** Leucopelargonidin **(F)** Kaempferol **(G)** Apigenin **(H)** Dihydrokaempferol **(I)** Genkwanin. Arrow indicates hydrogen bond.

Table S1 **Primer detail used in the study**.

Table S2 **List of interacting amino acid residues of CfCYP93B and CfCYP706C with different ligands**.

Table S3 **Analysis of drug like properties of ligands used in the study**.

File S1 **Method development for quantification of genkwanin**.
