# Corrigendum: Mannitol Stress Directs Flavonoid Metabolism toward Synthesis of Flavones via Differential Regulation of Two Cytochrome P450 Monooxygenases in *Coleus forskohlii*

**DOI:** 10.3389/fpls.2017.02222

**Published:** 2018-01-08

**Authors:** Praveen Awasthi, Ajai Prakash Gupta, Yashbir S. Bedi, Ram A. Vishwakarma, Sumit G. Gandhi

**Affiliations:** ^1^Indian Institute of Integrative Medicine (CSIR-IIIM), Council of Scientific and Industrial Research, Jammu, India; ^2^Quality Control, Quality Assurance & CMC Division, Council of Scientific and Industrial Research-Indian Institute of Integrative Medicine, Jammu, India; ^3^Division of Biological Science, Faculty of Science, Academy of Scientific and Innovative Research, Kolkata, India

**Keywords:** 7-O-methylapigenin, CYP93B, CYP706C, genkwanin, naringenin

In this published article, there was a labeling error in the graph-legend of Figure 3B. Correct labeling is: Red line and blue line stand for CfCYP706C and CfCYP93B respectively. Corrected Figure 3B is presented here.

**Figure 3:**
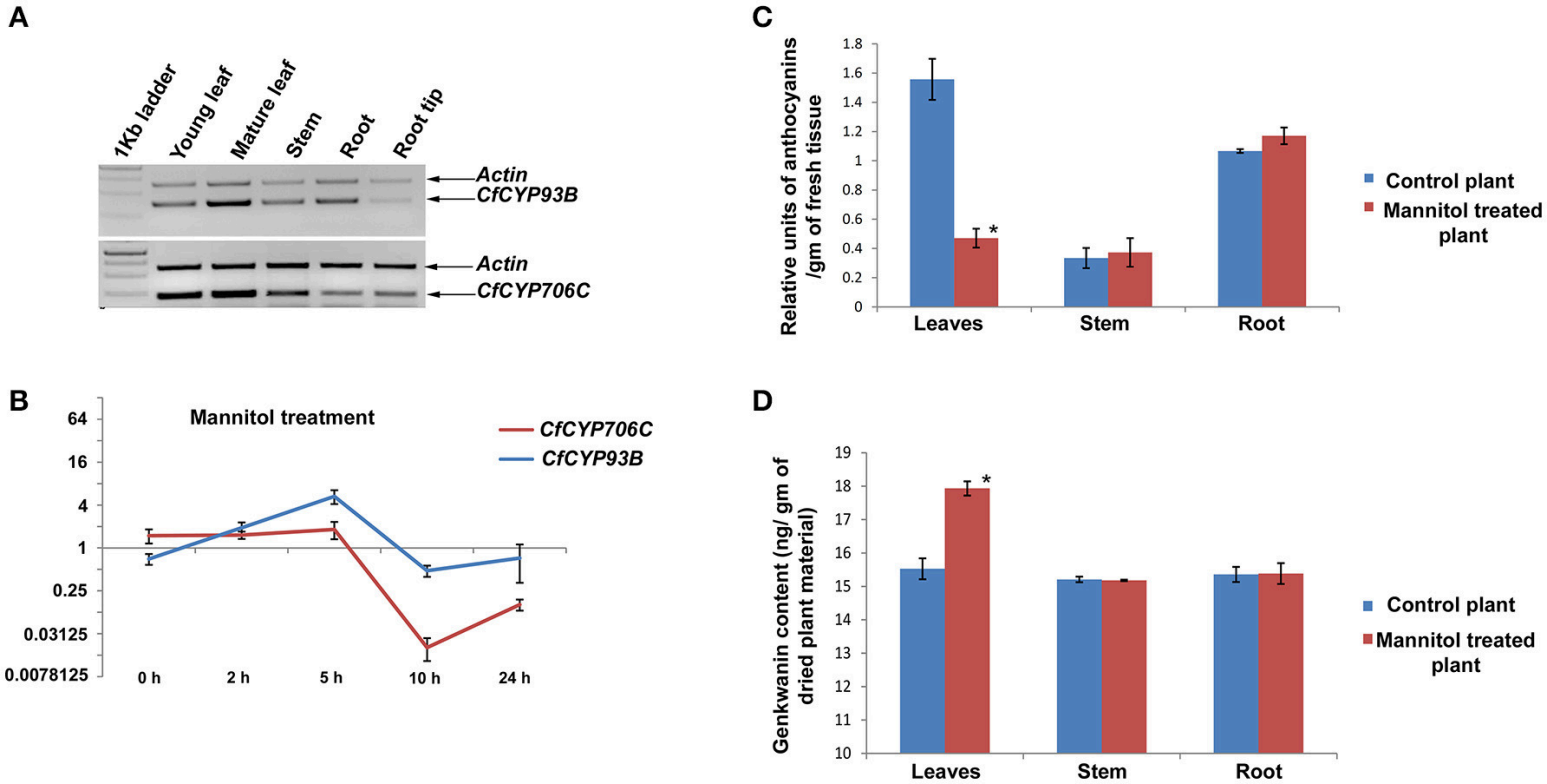
Expression study of *CfCYP93B* and *CfCYP706C* and its correlation with genkwanin and anthocyanin content. **(A)** Semi quantitative RT-PCR expression study of *CfCYP93B* and *CfCYP706C* in different tissues (young leaves, mature leaves, stems, roots and root tips) of *C. forskohlii* **(B)** qPCR study of *CfCYP93B* and *CfCYP706C* in response to mannitol treatment at diffetent time interval (0 h, 2 h, 5 h, 10 h and 24 h). Actin was used as housekeeping gene. For qPCR study, *p* < 0.05. **(C)** Relative anthocyanin units in different tissues (leaves, stem and root) in response to mannitol treatment. **(D)** Genkwanin content in different tissues (leaf, stem and root) in response to mannitol treatment. ^*^p < 0.05.

The authors apologize for this error and state that this does not change the scientific conclusions of the article in any way.

The original article has been updated.

## Conflict of interest statement

The authors declare that the research was conducted in the absence of any commercial or financial relationships that could be construed as a potential conflict of interest.

