# Supplementary figures and images for: Mannitol Stress Directs Flavonoid Metabolism toward Synthesis of Flavones via Differential Regulation of Two Cytochrome P450 Monooxygenases in Coleus forskohlii
Source: Front Plant Sci. 2016 Jul 6;7:985. doi: 10.3389/fpls.2016.00985 (PMC4933719; doi:10.3389/fpls.2016.00985)

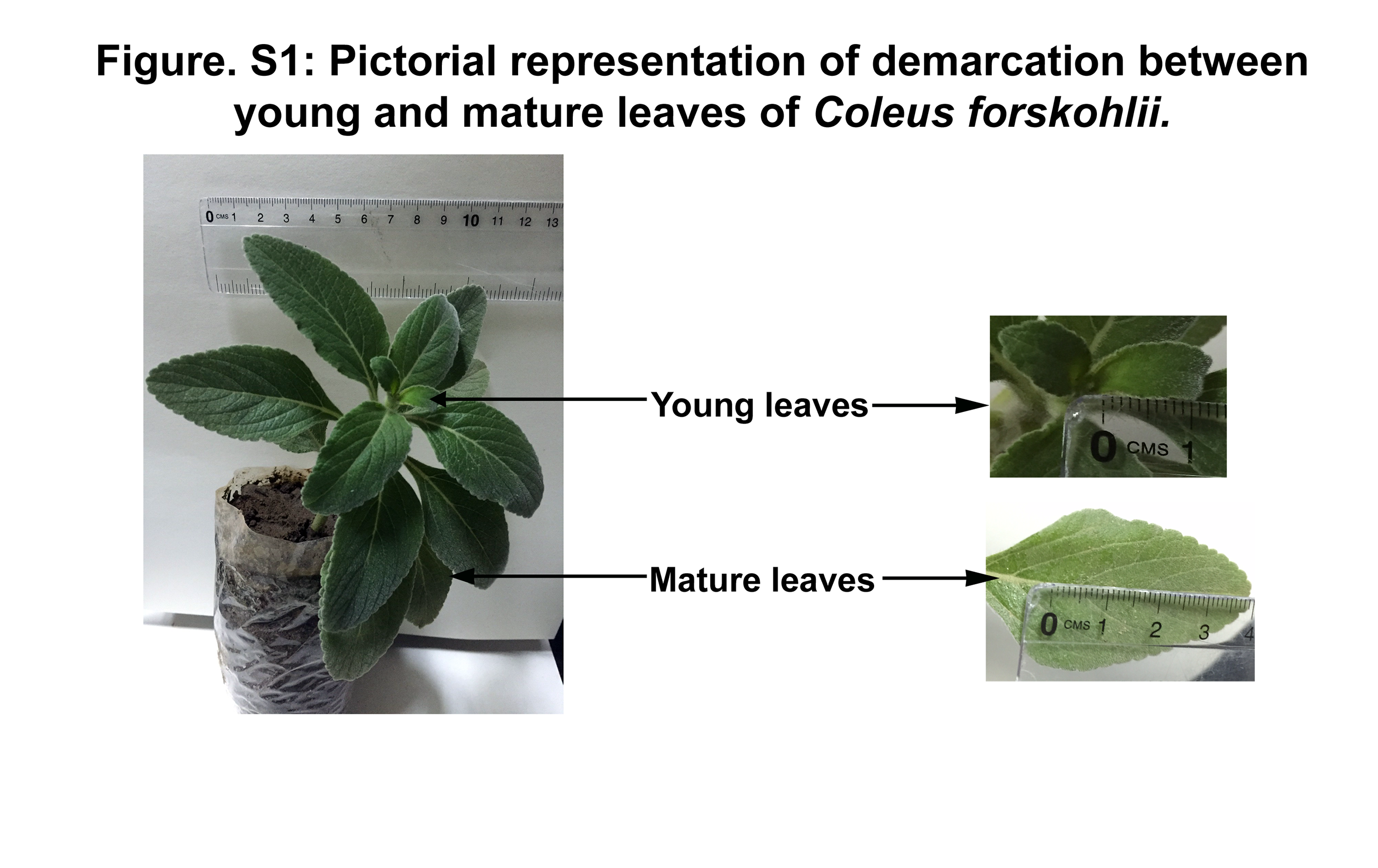

Supplement: Figure S1 — Pictorial representation of demarcation between young and mature leaves of Coleus forskohlii. [file Image1.TIF]

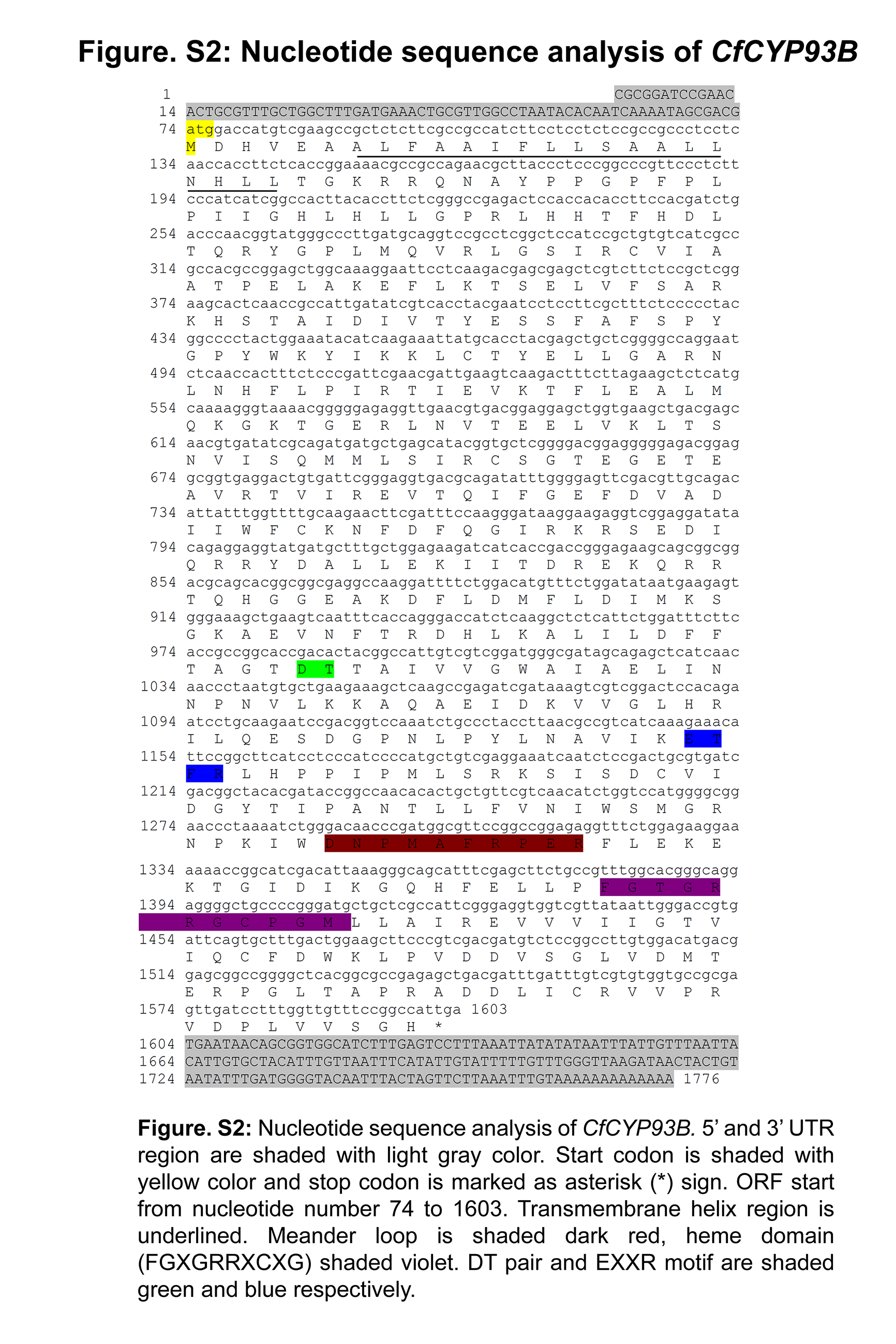

Supplement: Figure S2 — Nucleotide sequence analysis of CfCYP93B. 5′ and 3′ UTR region are shaded with light gray color. Start codon is shaded with yellow color and stop codon is marked as asterisk (*) sign. Transmembrane helix region is underlined. ORF start from nucleotide number 74–1603. Meander loop is shaded dark red, heme domain (FGXGRRXCXG) shaded violet. DT pair and EXXR motif are shaded green and blue, respectively. [file Image2.TIF]

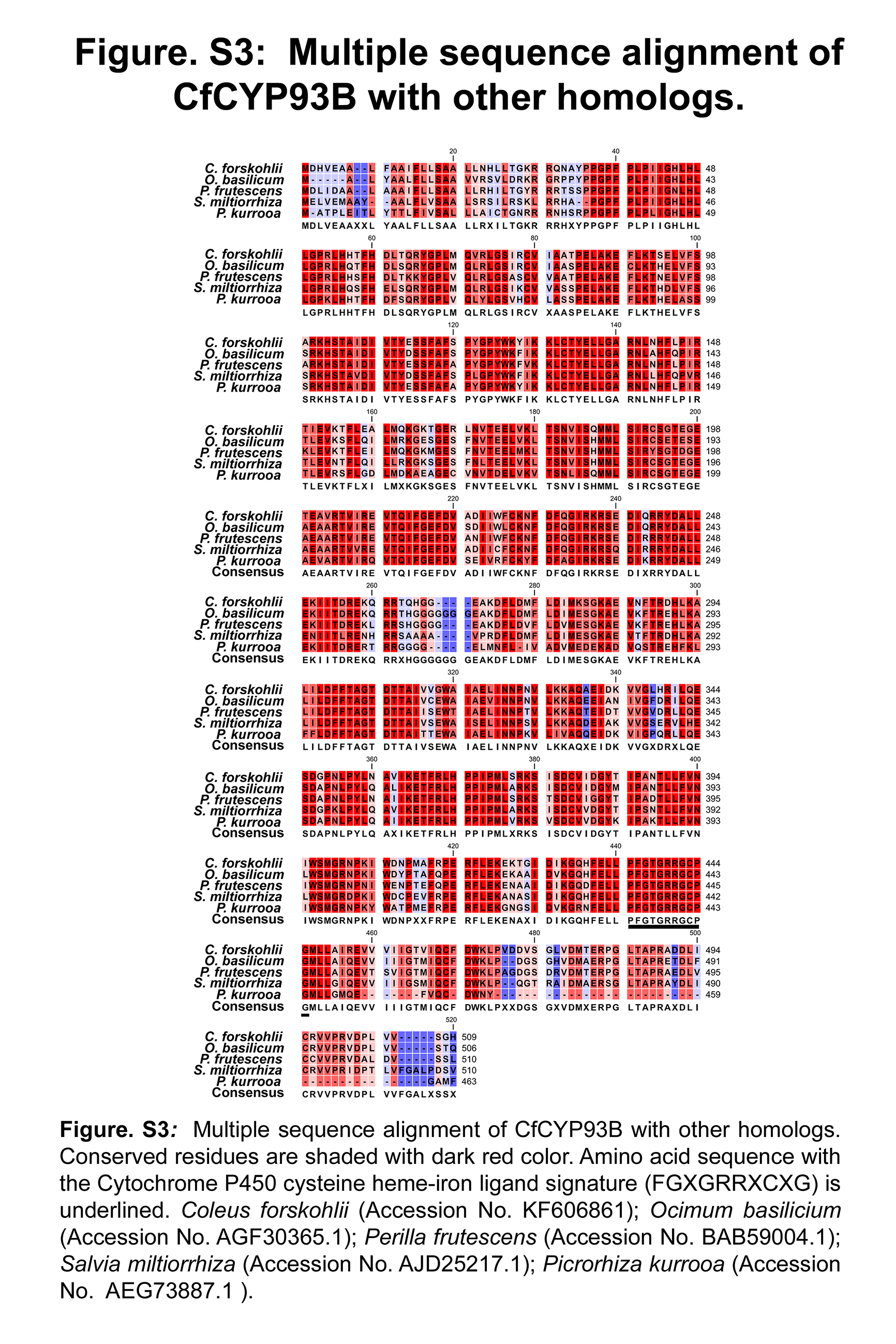

Supplement: Figure S3 — Multiple sequence alignment of CfCYP93B with other homologs. Conserved residues are shaded with dark red color. Amino acid sequence with the Cytochrome P450 cysteine heme-iron ligand signature (FGXGRRXCXG) is underlined. Coleus forskohlii (Accession No. KF606861); Ocimum basilicium (Accession No. AGF30365.1); Perilla frutescens (Accession No. BAB59004.1); Salvia miltiorrhiza (Accession No. AJD25217.1); Picrorhiza kurrooa (Accession No. AEG73887.1). [file Image3.TIF]

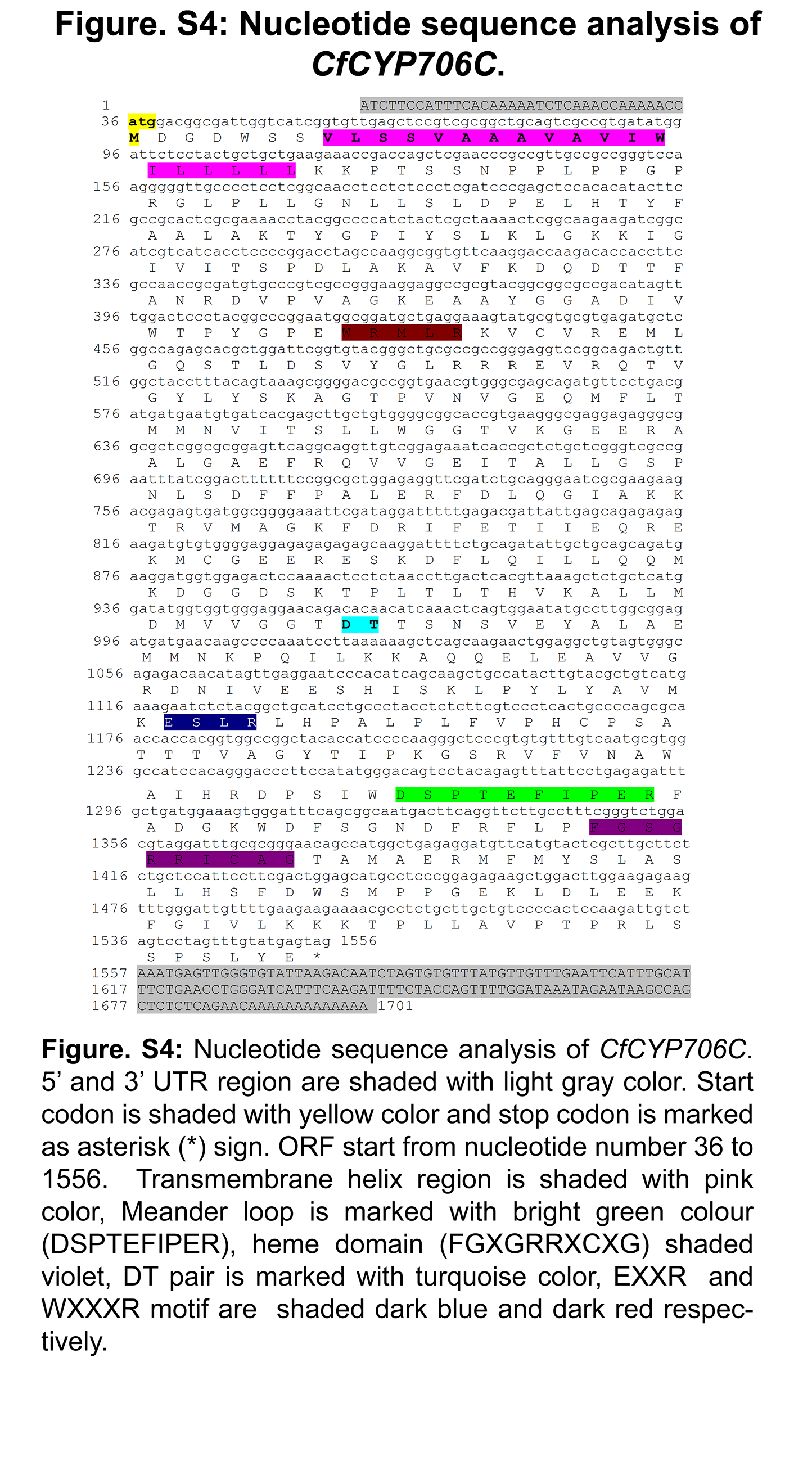

Supplement: Figure S4 — Nucleotide sequence analysis of CfCYP706C. 5′ and 3′ UTR region are shaded with light gray color. Start codon is shaded with yellow color and stop codon is marked as asterisk (*) sign. ORF start from nucleotide number 36–1556. Transmembrane helix region is shaded with pink color, Meander loop is marked with bright green color (DSPTEFIPER), heme domain (FGXGRRXCXG) shaded violet, DT pair is marked with turquoise color, EXXR, and WXXXR motif are shaded dark blue and dark red, respectively. [file Image4.TIF]

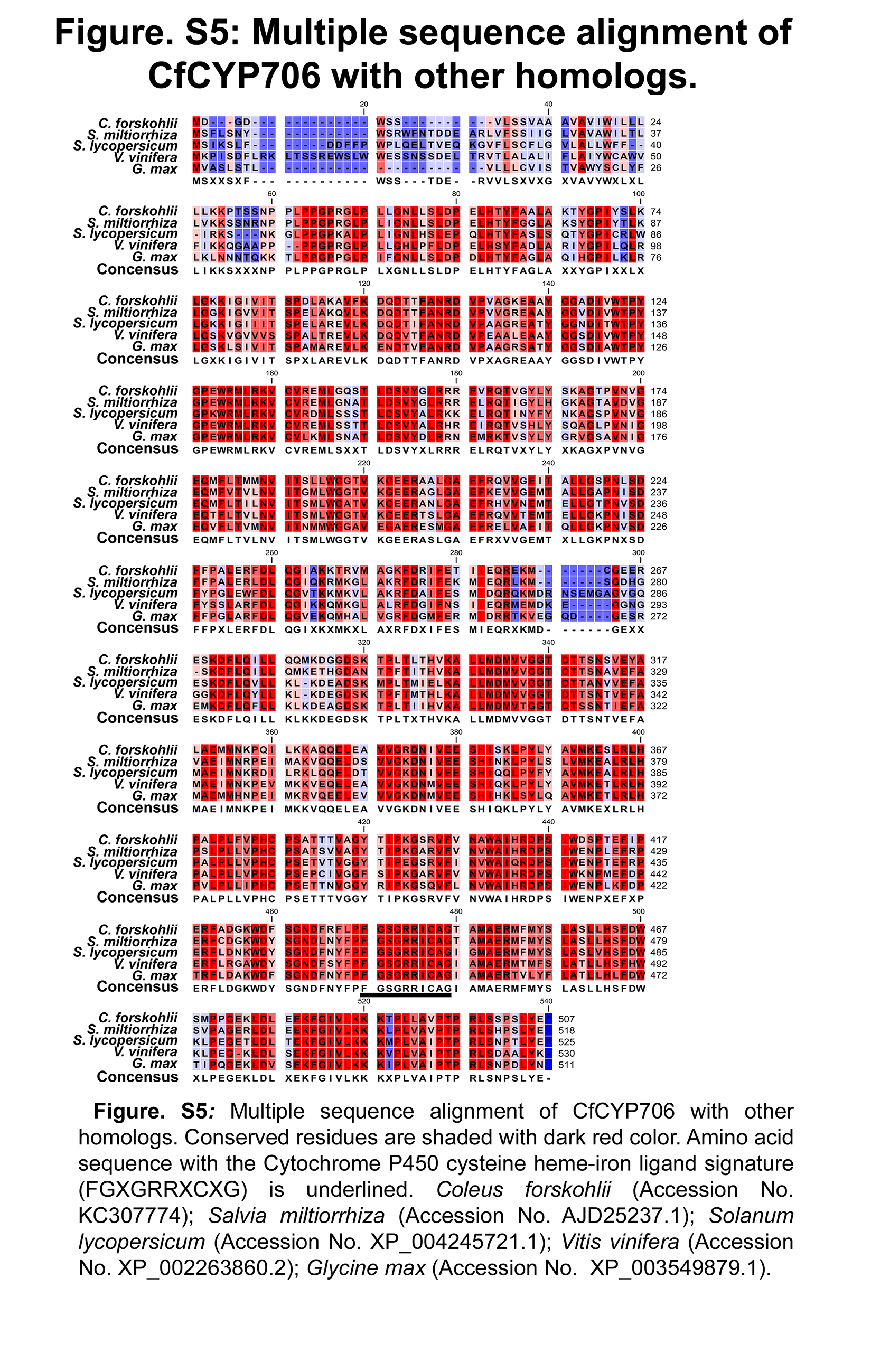

Supplement: Figure S5 — Multiple sequence alignment of CfCYP706 with other homologs. Conserved residues are shaded with dark red color. Amino acid sequence with the Cytochrome P450 cysteine heme-iron ligand signature (FGXGRRXCXG) is underlined. Coleus forskohlii (Accession No. KC307774); Salvia miltiorrhiza (Accession No. AJD25237.1); Solanum lycopersicum (Accession No. XP_004245721.1); Vitis vinifera (Accession No. XP_002263860.2); Glycine max (Accession No. XP_003549879.1). [file Image5.TIF]

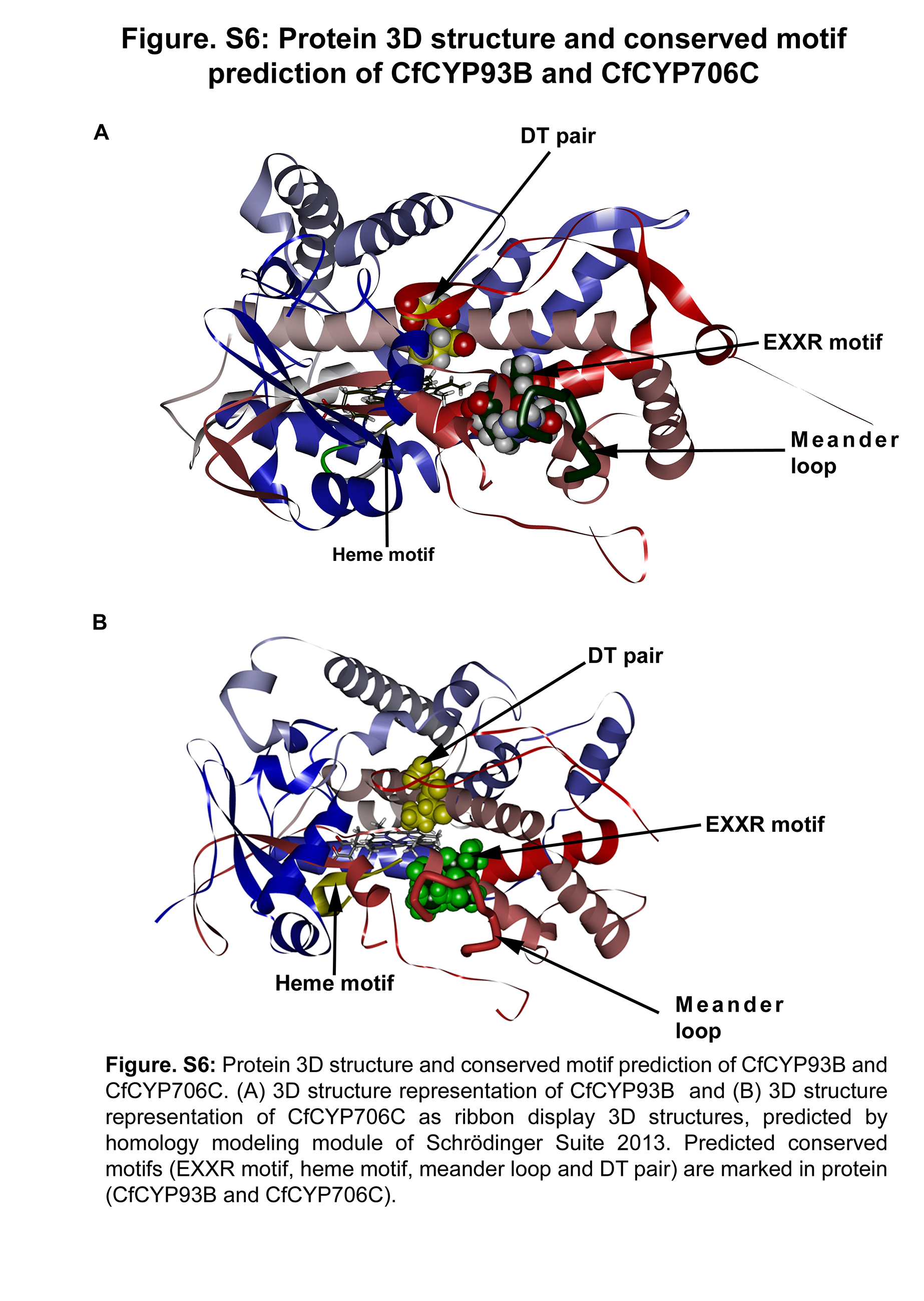

Supplement: Figure S6 — Protein 3D structure and conserved motif prediction of CfCYP93B and CfCYP706C. (A) 3D structure representation of CfCYP93B and (B) 3D structure representation of CfCYP706C as ribbon display 3D structures, predicted by homology modeling module of Schrödinger Suite 2013. Predicted conserved motifs (EXXR motif, heme motif, meander loop, and DT pair) are marked in protein (CfCYP93B and CfCYP706C). [file Image6.TIF]

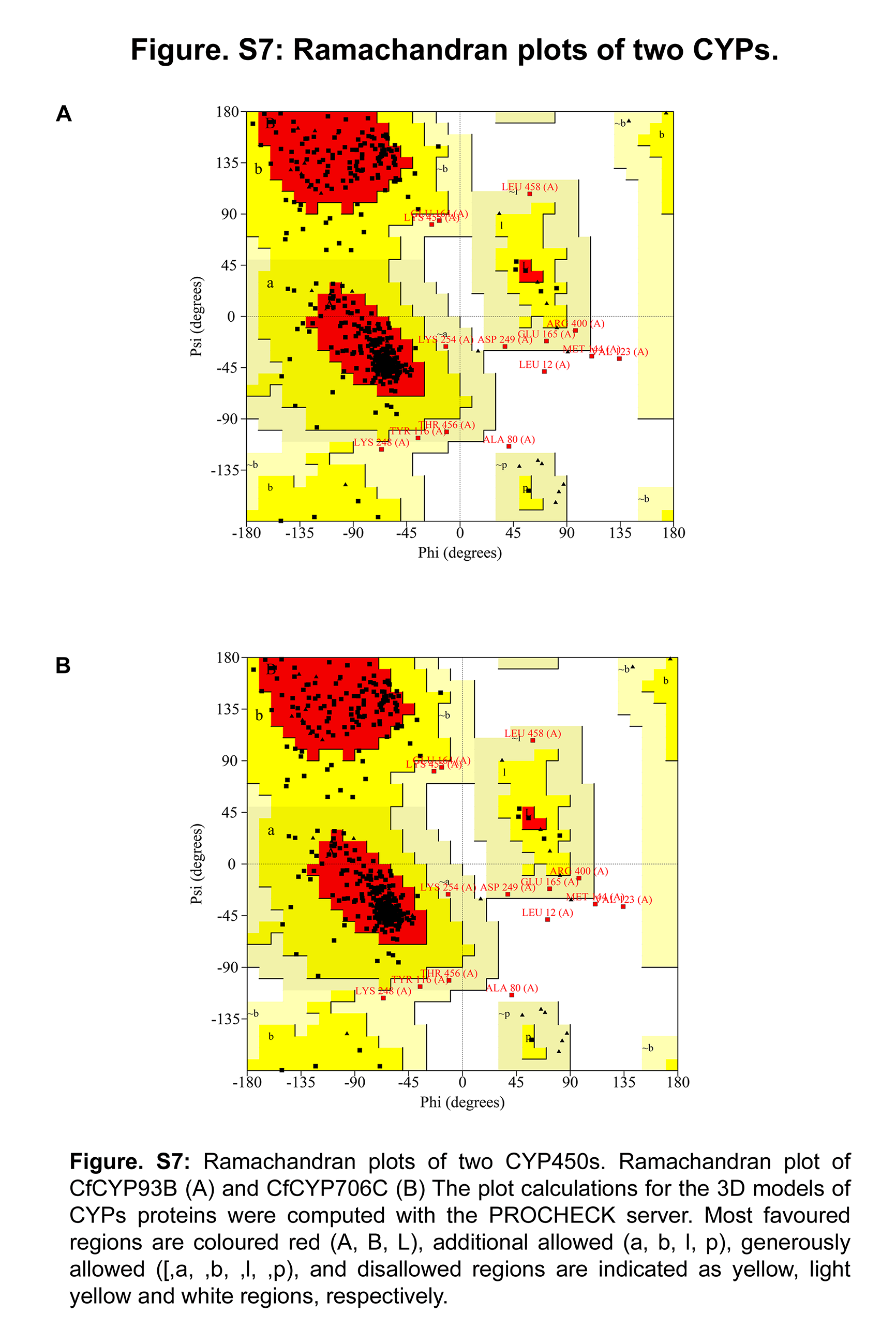

Supplement: Figure S7 — Ramachandran plot of two CYPs. Ramachandran plot of CfCYP93B (A) and CfCYP706C (B). The plot calculations for the 3D models of CYPs proteins were computed with the PROCHECK server. Most favored regions are colored red (A, B, L), additional allowed (a, b, l, p), generously allowed [,a, ,b, ,l, ,p), and disallowed regions are indicated as yellow, light yellow, and white regions, respectively. [file Image7.TIF]

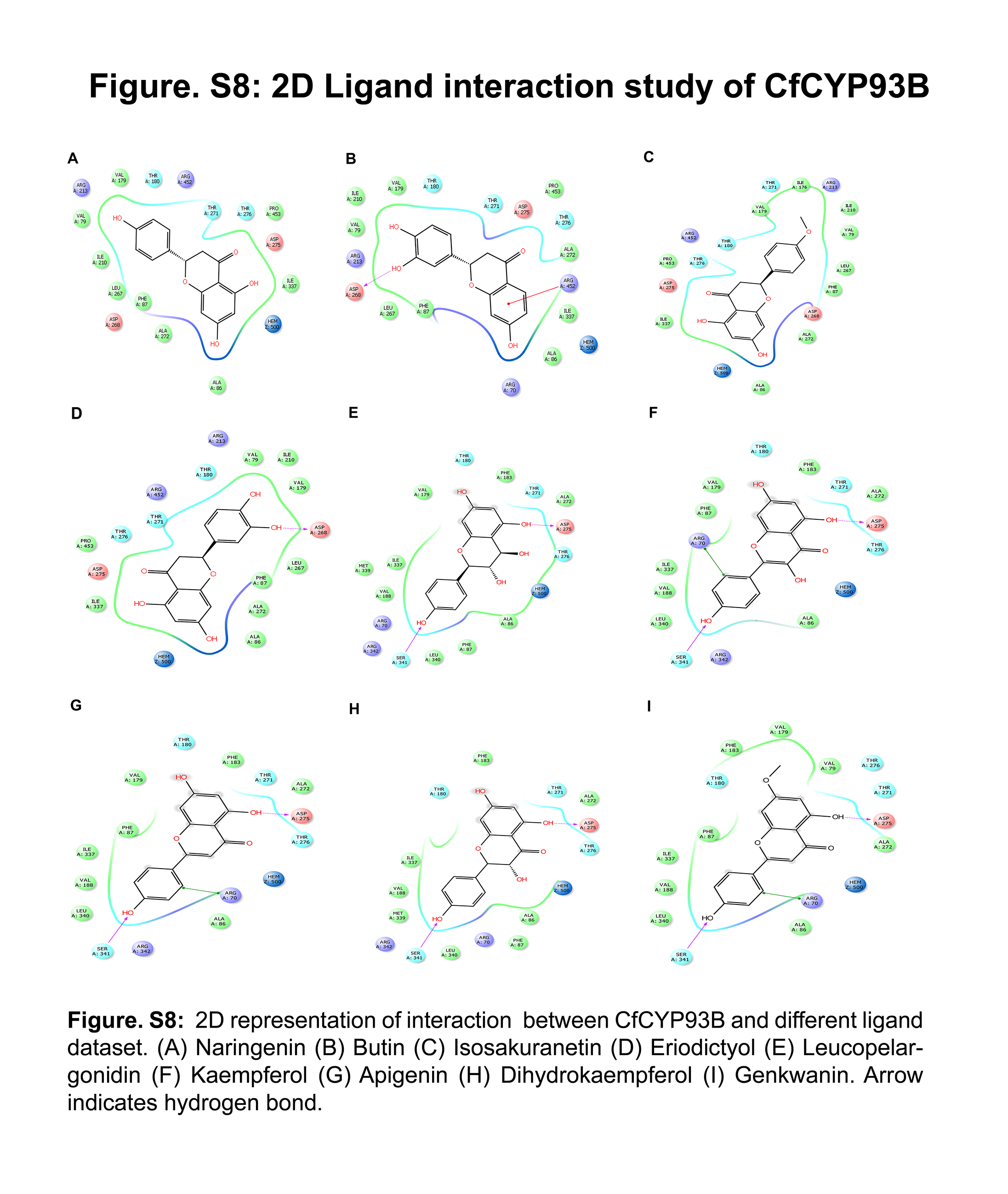

Supplement: Figure S8 — 2D Representation of interaction between CfCYP93B and different ligand dataset. (A) Naringenin, (B) Butin, (C) Isosakuranetin, (D) Eriodictyol, (E) Leucopelargonidin, (F) Kaempferol, (G) Apigenin, (H) Dihydrokaempferol, (I) Genkwanin. Arrow indicates hydrogen bond. [file Image8.TIF]

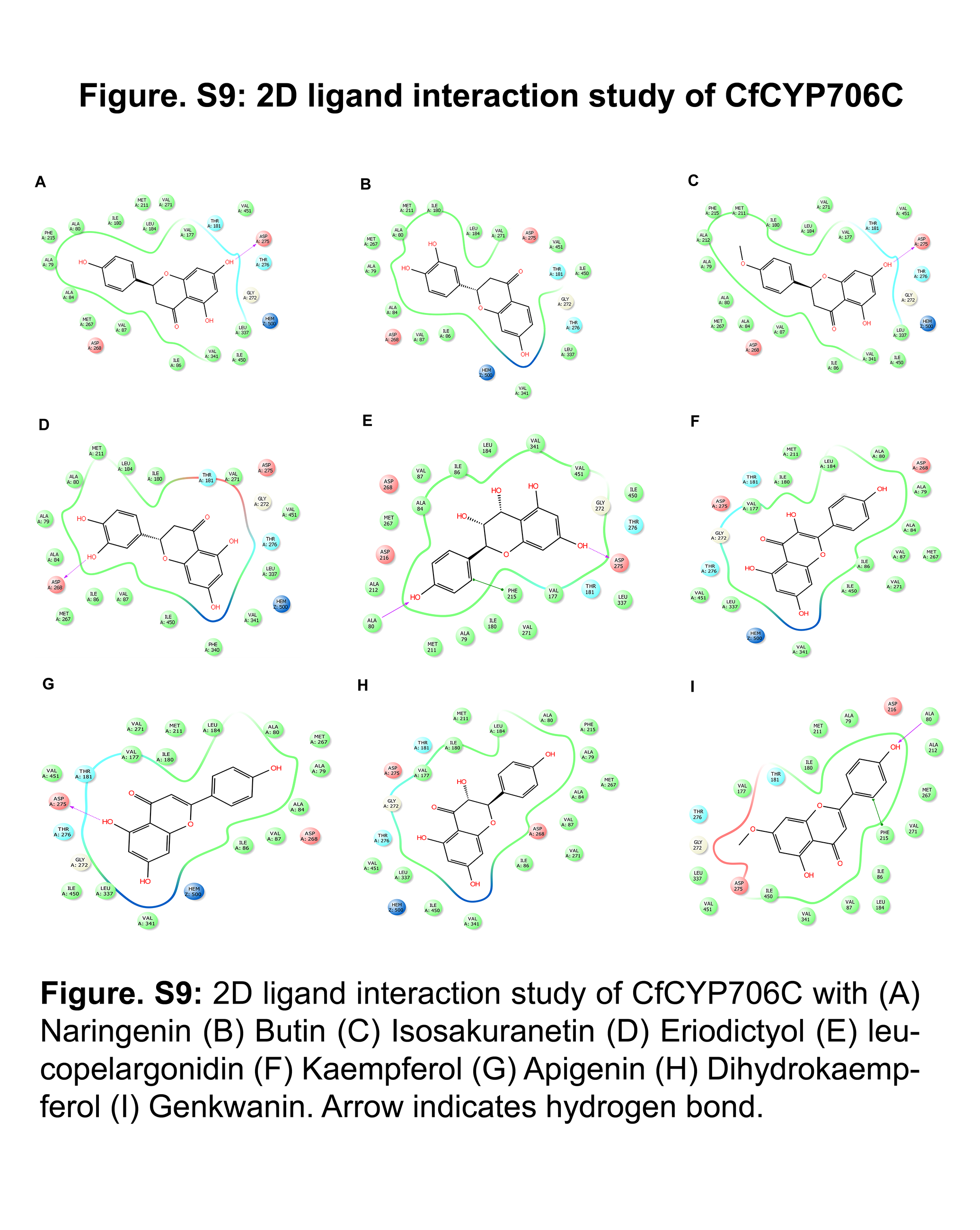

Supplement: Figure S9 — 2D Representation of interaction between CfCYP706C different ligand dataset. (A) Naringenin (B) Butin (C) Isosakuranetin (D) Eriodictyol (E) Leucopelargonidin (F) Kaempferol (G) Apigenin (H) Dihydrokaempferol (I) Genkwanin. Arrow indicates hydrogen bond. [file Image9.TIF]
