# Supplementary material for: Mannitol Stress Directs Flavonoid Metabolism toward Synthesis of Flavones via Differential Regulation of Two Cytochrome P450 Monooxygenases in Coleus forskohlii
Source: Front Plant Sci. 2016 Jul 6;7:985. doi: 10.3389/fpls.2016.00985 (PMC4933719; doi:10.3389/fpls.2016.00985)
Supplement: Table S1 — Primer detail used in the study. [file Table1.DOCX]

**Table S1:** Primer used in the study

| **Primer Name** | **Sequence (5’-----> 3’)** | **T_m_ (°C)** | **Orientation** | **Primer used for:** |
| --- | --- | --- | --- | --- |
| D1 | GCGGATCCGARGARTTYMGNCCNGAGAG | 75.5 | Forward | Used for primary PCR for differential display study |
| FG1 | CGCCATTTGG | 41.8 | Forward | Used for secondary (nested) PCR for differential display study |
| FG2 | CGCCATTCGG | 46.3 | Forward |  |
| FG5 | CGCCCTTCGG | 46.5 | Forward |  |
| 3' RACE Adapter | GCGAGCACAGAATTAATACGACTCACTATAGGT12VN | 45 | Reverse | Used for cloning of CfCYP93B and CfCYP706C gene |
| 5' RACE Outer | GCTGATGGCGATGAATGAACACTG | 55 | Forward |  |
| 5' RACE Inner | CGCGGATCCGAACACTGCGTTTGCTGGCTTTGATG | 55 | Forward |  |
| 3' RACE Outer | GCGAGCACAGAATTAATACGACT | 45 | Reverse |  |
| GSPCfCYP93B5R | CCACACGACAAATCAAATCG | 55 | Reverse |  |
| GSPCfCYP706C5R | ATGGAGCAGAGAAGCAAGCGAGT | 55 | Reverse |  |
| cdsCfCYP93BF | ATGGACCATGTCGAAGCCGCTCTC | 55 | Forward |  |
| cdsCfCYP93BR | TCAATGGCCGGAAACAACCAAAGG | 55 | Reverse |  |
| cdsCfCYP706CF | ATGGACGGCGATTGGTCATCGGTG | 55 | Forward |  |
| cdsCfCYP706CR | CTACTCATACAAACTAGGACTAGAC | 55 | Reverse |  |
| RTCfCYP93B1F | CCACACGACAAATCAAATCG | 60 | Forward | Used for Expression study |
| RTCfCYP93B1R | ATCGACGGCTACACGATACC | 64 | Reverse |  |
| RTCfCYP706C1F | CTCCCGTGTGTTTGTCAATG | 55 | Forward |  |
| RTCfCYP706C1R | ATGGAGCAGAGAAGCAAGCGAGT | 55 | Reverse |  |
| RTactinF | CCGTGGAGAAGAGCTACGAG | 56.9 | Forward | *Actin* gene; used as housekeeping control in expression study |
| RTactinR | TCACACTTCATGATGGAGTTGTAGG | 56.5 | Reverse |  |
| M13F | GTAAAACGACGGCCAGT | 52.6 | Forward | Used for amplification of insert in pTZ57R/T vector |
| M13R | CAGGAAACAGCTATGAC | 47 | Reverse |  |
