# Supplementary material for: Mannitol Stress Directs Flavonoid Metabolism toward Synthesis of Flavones via Differential Regulation of Two Cytochrome P450 Monooxygenases in Coleus forskohlii
Source: Front Plant Sci. 2016 Jul 6;7:985. doi: 10.3389/fpls.2016.00985 (PMC4933719; doi:10.3389/fpls.2016.00985)
Supplement: Table S2 — List of interacting amino acid residues of CfCYP93B and CfCYP706C with different ligands. [file Table2.DOCX]

| **S.No** | **Metabolite** | **Interacting Residues** | |
| --- | --- | --- | --- |
|  |  | **CfCYP93B** | **CfCYP706C** |
| 1 | Naringenin | ARG213, VAL179, THR190, ARG452, THR271, THR276, PRO453, ASP275, ILE337, HEM500, ALA86, ALA272, PHE87, ASP268, LEU267, ILE210 and VAL79 | VAL177, THR181, VAL451, ASP275, THR276, GLY272, HEM500, LEU337, ILE450, VAL341, ILE86, VAL87, ASP268, MET267, ALA84, ALA79, PHE215, ALA80, ILE180, LEU184, MET211 and VAL271 |
| 2 | Isosakuranetin | ARG452, THR180, VAL179, THR271, ILE176, ARG213, ILE210, VAL79, LEU267, PHE87, ASP268, ALA272, ALA86, HEM500, ILE337, ASP275, PRO453 and THR276 | MET211, ILE180, LEU184, VAL271, VAL177, THR181, VAL451, ASP275, THR276, GLY272, HEM500, LEU337, ILE450, VAL341, ILE86, VAL87, ASP268, ALA84, MET627, ALA80, ALA79, ALA212 and PHE215 |
| 3 | Eriodictyol | AGR213, VAL79, ILE210, VAL179, ASP268, LEU267,PHE87, ALA272, ALA89, HEM500, ILE337, ASP275, PRO453, THR276,THR271, ARG452 and THR180 | MET211, LEU184, ILE180, THR181, VAL271, ASP275, GLY272, VAL451, THR276, LEU337, HEM500, VAL341, PHE340, ILE450, VAL87, ILE86, MET267, ASP268, ALA84, ALA79 and ALA80 |
| 4 | Butin | ILE210, VAL179, THR180, THR271, ASP275, PRO453, THR276, ALA272, ARG452, ILE337, HEM500, ALA86, PHE87, LEU267, ASP268, ARG213 and VAL79 | MET211, ILE180, LEU184, VAL271, ASP275, VAL451, THR181, ILE450, GLY272, THR276, LEU337, VAL341, HEM500, ILE86, VAL87, ASP268, ALA84, ALA79, MET267 and ALA80 |
| 5 | Genkwanin | THR180, PHE183, VAL179, VAL79, THR276, THR271, ASP275, ALA272, HEM500, ARG70, ALA86, SER341, LEU340, VAL188, ILE337and PHE87 | MET211, ALA79, ASP216, ALA80, ALA212, MET267, VAL271, PHE215, ILE86, LEU184, VAL87, VAL341, ILE450, ASP275, VAL451, LEU337, GLY272, THR276, VAL177, THR181 and ILE180 |
| 6 | Apigenin | VAL179, THR180, PHE183, THR271, ALA272, ASP275, THR276, HEM500, ARG70, ALA86, ARG342, SER341, LEU340, VAL188, ILE337 and PHE87 | ASP275, VAL451, THR181, VAL177, VAL271, ILE180, MET211, LEU184, ALA80, MET267, ALA79, ALA84, ASP268, VAL87, ILE86, HEM500, VAL341, LEU337, ILE450, GLY272 and THR276 |
| 7 | Leucopelargonidin | VAL179, THR180, PHE183, THR271, ALA272, ASP275, THR276, HEM500, ALA86, PHE87, LEU340, SER341, ARG341, ARG342, ARG70, VAL188, MET339 and ILE337 | MET267, ALA84, ASP268, VAL87, ILE86, LEU184, VAL341, VAL451, GLY272, ILE450, THR276, ASP275, THR181, LEU337, VAL177, PHE215, VAL271, ILE180, ALA79, MET211, ALA80, ALA212 and ASP216 |
| 8 | Dihydrokaempferol | THR180,PHE183, THR271, ALA272, ASP275, THR276, HEM500, ALA86, PHE87, ARG70, LEU340, SER341, ARG342, MET339, VAL188 and ILE337 | MET211, LEU184, ALA80, PHE215, ALA79, MET267, ALA84, VAL87, ASP268, VAL271, ILE86, VAL341, ILE450, HEM500, LEU337, VAL451, THR276, GLY272, ASP275, VAL177, THR181 and ILE180 |
| 9 | Kaempferol | VAL179, THR180, PHE183, THR271, ALA272, ASP275, THR276, HEM500, ALA86, ARG342, SER341, LEU340, VAL188, ILE337, ARG70 and PHE87 | MET211, LEU184, ALA80, ASP268, ALA79, ALA84, VAL87, MET267, ILE86, VAL271, ILE450, VAL341, HEM500, VAL177, THR188 and ILE180 |

**Table S2**: List of amino acid residues of CfCYP93B and CfCYP706C that interact with different ligands.
