# Supplementary material for: Mannitol Stress Directs Flavonoid Metabolism toward Synthesis of Flavones via Differential Regulation of Two Cytochrome P450 Monooxygenases in Coleus forskohlii
Source: Front Plant Sci. 2016 Jul 6;7:985. doi: 10.3389/fpls.2016.00985 (PMC4933719; doi:10.3389/fpls.2016.00985)
Supplement: Table S3 — Analysis of drug like properties of ligands used in the study. [file Table3.DOCX]

| **S. No** |  | **Mass** | **Hydrogen bond donor** | **Hydrogen bond acceptor** | **LogP** | **Molar refractivity** |
| --- | --- | --- | --- | --- | --- | --- |
| 1 | Naringenin | 272 | 3 | 5 | 2.50900 | 70.194893 |
| 2 | Isosakuranetin | 286 | 2 | 5 | 2.812899 | 75.082085 |
| 3 | Eriodictyol | 288 | 4 | 6 | 2.21550 | 71.859695 |
| 4 | Butin | 272 | 3 | 5 | 2.50900 | 70.194893 |
| 5 | Genkwanin | 284 | 2 | 5 | 2.722599 | 75.701080 |
| 6 | Apigenin | 270 | 0 | 5 | 1.488870 | 61.368496 |
| 7 | Leucopelargonidin | 290 | 5 | 6 | 1.331400 | 72.213989 |
| 8 | Dihydrokaempferol | 288 | 4 | 6 | 1.480700 | 71.584694 |
| 9 | Kaempferol | 286 | 1 | 6 | 1.189070 | 62.247295 |

**Table S3:** Analysis of drug like properties of ligands used in the study
