# Supplementary material for: Mannitol Stress Directs Flavonoid Metabolism toward Synthesis of Flavones via Differential Regulation of Two Cytochrome P450 Monooxygenases in Coleus forskohlii
Source: Front Plant Sci. 2016 Jul 6;7:985. doi: 10.3389/fpls.2016.00985 (PMC4933719; doi:10.3389/fpls.2016.00985)

**File S1: Method development for quantification of genkwanin**

An LC-ESI-MS/MS method through MRM (multiple reaction monitoring) was developed for the selective detection and quantification of genkwanin at 11.4 min in the extract. The selectivity and sensitivity of (MRM) LC-ESI-MS/MS rely on the application of MRM in which each ionized compound gives a distinct precursor-to-product ion transition that is diagnostic for the presence of a particular compound in an extract. Comparison of the chromatograms of blank and spiked standard substance indicated no significant interference at the retention times of the investigated compounds. In the optimized method, Chromolith RP18e column (100 X4.6 mm) was used for optimal separation of genkwanin. The presence of genkwanin was successfully detected by mass fragmentography using two MRM transitions. Standards were injected using LC without column (through the union) controlled by Mass-Hunter workstation software ver. B.04.00, for qualitative optimization.

Chromatograms of MRM transition mass of 283/268 for genkwanin, (Fig. S9). As seen in the figure, ions peaks were sufficiently separated and, genkwanin was present in all the samples. Notably, the present method reports for the first time use of Chromolith C-18e column and (MRM) LC-ESI-MS/MS for the separation and quantification of genkwanin. The developed multimode LC-ESI-MS/MS method can easily be utilized as a fast and sensitive analytical tool for analysis of genkwanin in the presence of other possible constituents.

Chromatographic development

Liquid chromatography analyzes were carried out using a 1260 Infinity quaternary pump equipped with an auto sampler, column heater and online degasser. A Chromolith C18e column (100 X4.6 mm) protected by a guard column at 30^0^C was used for analytical chromatographic separations of extract. Samples were injected (20 µl) in triplicate. The elution was carried out in a binary gradient solvent system consisting of H_2_O with 0.1% formic acid (solvent A) and Acetonitrile (solvent B). The flow rate was optimized to 0.6 mL/min. Gradient elution was programmed as follows: 0.0 min, 30% B; 0–8 min, 30–50% B; 8–12 min, 50-70% B; 12–13 min, 70–30% B; and 13-15 min, 30% B.

Spectra generated for genkwanin (m/z 283.0) in negative ion detection gave the protonated molecule [M-H]^-^. During MRM method development, fragmentor voltage for the maximum abundance of genkwanin [M-H]^-^and collision energy for the generation of product ion were optimized to get a good signal of fragmented ions. Maximum resolution was obtained at fragmentor voltage of 140 V and collision energy of 10 eV for genkwanin. Quantification of genkwanin in the extract was done by injecting the extracted sample in the MRM mode. Identification of genkwanin was done on the basis of retention time and comparison of the presence of a peak in the MRM and the standard.

Mass spectrometric conditions optimization

The experiment was conducted in both polarities (positive and negative) of APCI and ESI mode. The response was found to be better in negative ESI. For optimization of spectrometric conditions, the range of fragmentor voltage (50–180 V), capillary voltage (2500–3500 V), collision energy (5–50 eV) and sensitivity for the fragmented ions of genkwanin studied compounds were investigated.

Preparation of calibration curves for standard

To begin with one blank injection was run to check the noise level of the system. Stock solution (1mg/ml) was appropriately diluted for making five point calibrations curve for genkwanin. The calibration equation of components were detected using LC-electrospray ionization (ESI) in negative-ion mode and quantified by Chromatograms of MRM transition mass of 283/268 for genkwanin, were obtained by plotting LC–MS peak area (y) versus the concentration (x, ng/ml) of calibrators y = 1021.473342x- 15447.969250 (r^2^ = 0.991). The equation showed very good linearity over the range.


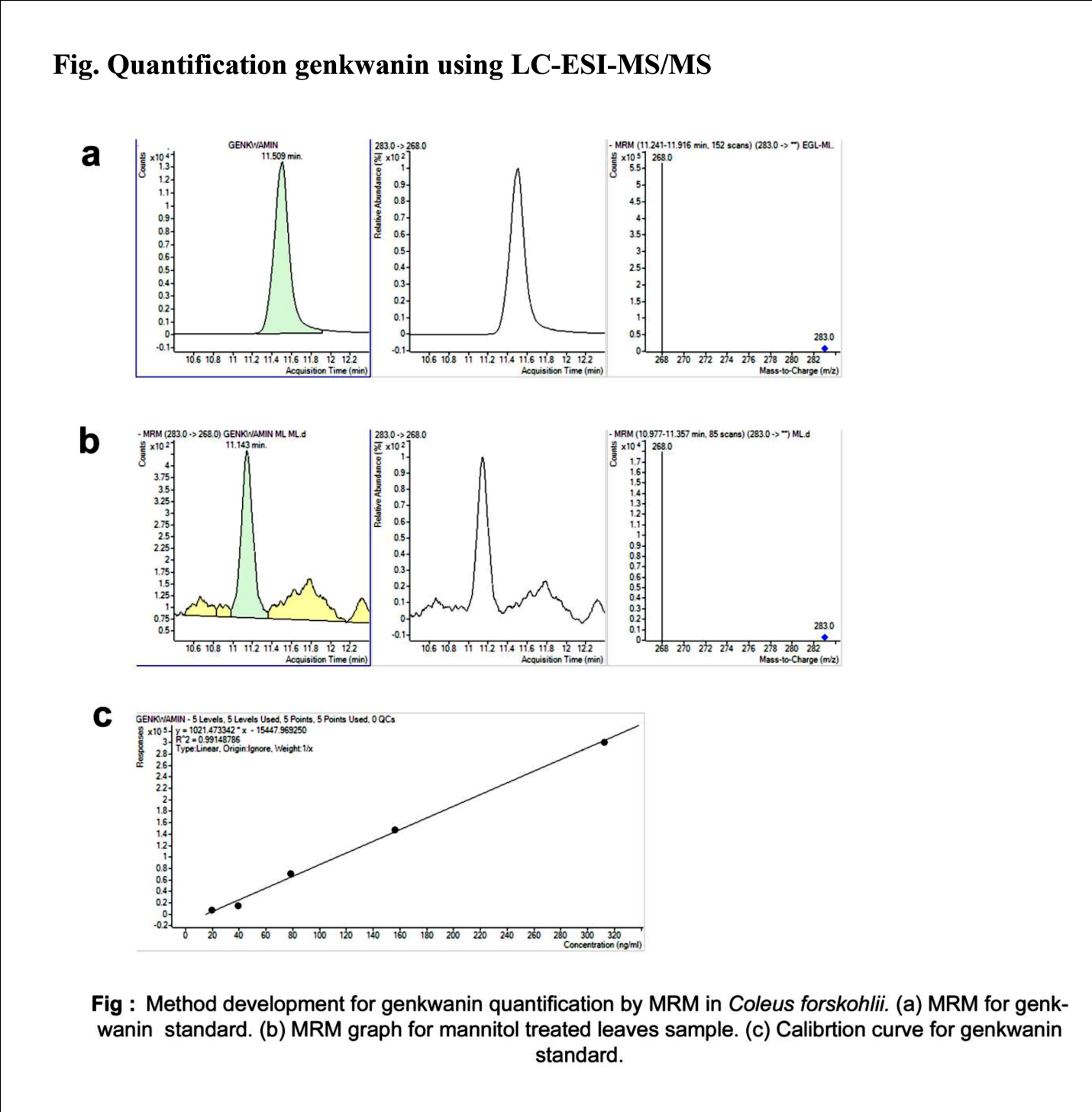

Supplement: File S1 — Method development for quantification of genkwanin. [file DataSheet1.docx]
